# Virion-Independent Extracellular Vesicle (EV)-Dependent Transmission of SARS-CoV-2 as a Potential New Mechanism of Viral RNA Spread in Human Cells

**DOI:** 10.3390/v18010145

**Published:** 2026-01-22

**Authors:** Nergiz Ekmen, Ali Riza Koksal, Dong Lin, Di Tian, Paul Thevenot, Sarah Glover, Srikanta Dash

**Affiliations:** 1 Division of Gastroenterology and Hepatology, Tulane University Health Sciences Center, New Orleans, LA 70112, USA; nekmen@tulane.edu (N.E.); sglover3@tulane.edu (S.G.); 2 Department of Pathology and Laboratory Medicine, Tulane University Health Sciences Center, 1430 Tulane Avenue, New Orleans, LA 70112, USA; akoksal@tulane.edu (A.R.K.); dlin6@tulane.edu (D.L.); dtian2@tulane.edu (D.T.); 3 Department of Gastroenterology and Hepatology, Institute of Translational Research, Ochsner Health, New Orleans, LA 70121, USA; paul.thevenot@ochsner.org; 4 Southeast Louisiana Veterans Health Care System, 2400 Canal Street, New Orleans, LA 70119, USA

**Keywords:** SARS-CoV-2, extracellular vesicles (EVs), subgenomic replicon, syncytia, double membrane vesicle (DMV)

## Abstract

The concentration of extracellular vesicles (EVs) in the peripheral blood of COVID-19 patients is increased. Nevertheless, their potential role in the transmission of infection remains unclear. This study was performed to determine whether EVs produced by the sub-genomic replicon system developed in Baby Hamster Kidney (BHK-21) cells could transfer SARS-CoV-2 replicon RNA, leading to the establishment of a viral replication system in human cells. Purified EVs from the SARS-CoV-2 sub-genomic replicon cell line BHK-21 were cultured with a naive human cell line. The success of EV-mediated transfer of SARS-CoV-2 replicon RNA and its productive replication was assessed using G-418 selection, a luciferase assay, immunostaining, and Western blot. We found that the A549 cell line cultured with EVs isolated from SARS-CoV-2 BHK-21 replicon cells developed G-418-resistant cell colonies. SARS-COV-2 RNA replication in A549 cells was confirmed by nano luciferase, Nsp1 protein. SARS-CoV-2 RNA replication causes massive morphological changes. Treatment of cells with the FDA-approved Paxlovid demonstrated a dose-dependent inhibition of viral replication. We isolated two human epithelial cell lines (gastrointestinal and neuroblastoma) and one vascular endothelial cell line that stably support high-level replication of SARS-CoV-2 sub-genomic RNA. Viral elimination did not revert the abnormal cellular shape, vesicle accumulation, syncytia formation, or EV release. Our study’s findings highlight the potential implications of EV-mediated transfer of replicon RNA to permissive cells. The replicon model is a valuable tool for studying virus-induced reversible and irreversible cellular reprogramming, as well as for testing novel therapeutic strategies for SARS-CoV-2.

## 1. Introduction

COVID-19 is an infectious disease caused by an RNA virus called severe acute respiratory syndrome coronavirus 2 (SARS-CoV-2) [1,2]. The COVID-19 pandemic killed millions of people worldwide. The primary mode of SARS-CoV-2 transmission was through inhaling air carrying respiratory fluids or aerosolized particles from an infected person. While most infected individuals experience mild-to-moderate illness and recover naturally, others can develop severe pneumonia that requires medical attention. Most severe diseases occur in older people with underlying medical conditions such as diabetes, obesity, cardiovascular disease, chronic respiratory disease, and cancer. In some individuals, COVID-19 can lead to long-lasting damage to the lungs, heart, kidneys, and nervous system, potentially leading to death. The potential for severe illness and complications linked to comorbidities and independent of age indicates that coronaviruses will likely remain a long-term threat to human public health [3]. The pathogenetic mechanisms linking virus replication, host tropisms, and viral dissemination from the lungs to other organs, such as the intestine, liver, kidneys, heart, spleen, and brain, remain poorly understood. SARS-CoV-2 infection disrupts both the epithelial and endothelial linings, promoting inflammation, coagulation, and immune cell infiltration, leading to an inflammatory cytokine storm. A detailed understanding of the virus–host interaction is needed to develop novel antiviral approaches to prevent mortality associated with any future coronavirus pandemic. Extracellular vesicles (EVs) shed during SARS-CoV-2 infection represent a potential mechanism that may explain various aspects of viral pathogenesis, including viral transmission, multi-organ dissemination, immune evasion, endothelial dysfunction, and systemic inflammatory responses and coagulopathy.

EVs are small membrane-derived vesicles continuously shed by various cells in the body to promote intracellular communication during both physiological and pathological conditions [4]. The budding of EVs directly from the cell membrane creates microvesicles (MVs) of about 0.5–1 μm in size. Vesicles derived from multivesicular bodies in the endo-lysosomal compartment are called exosomes, about 50–150 nm in size. EVs enter recipient cells by either direct membrane fusion or endocytosis. EVs in the peripheral blood carry heterogeneous cargo consisting of proteins, lipids, and various forms of nucleic acids. EVs in the tissue maintain cellular physiology and regulate hormone secretion, including insulin secretion from pancreatic islet cells in response to changes in blood glucose levels or inflammatory mediators [5]. EVs are also an essential component of the immune system, regulating aspects of both innate and adaptive immunity, including immune cell activation, antigen presentation, and immune modulation, while also contributing to the pathogenesis of various immune-related disorders [6]. Most importantly, EVs released from infected cells can exert immunomodulatory effects by modulating the host immune response to viruses, bacteria, fungi, or parasites, as well as by promoting vaccine escape, thereby leading to persistent infection and chronic disease [7].

A potential role for EVs in the transmission of RNA viruses could have significant therapeutic implications. Many viruses use the cellular membrane for EV production, cargo packaging, and secretion during the replication, assembly, and release phases. This establishes a direct connection between RNA virus replication and EV production [8,9,10]. EVs and viruses share overlapping structural and functional characteristics, making it difficult to distinguish between them. In a hepatitis C virus (HCV) cell culture model, we demonstrated that HCV replication enhances EV production, thereby persistently promoting virus infection throughout the culture [11]. Blocking EV release using a small molecule inhibited viral replication, suggesting that EV production is a primary mechanism for escaping the innate immune response. EVs also play a critical role in escaping the humoral antibody response. Hepatitis A virus particles released from infected cells are encapsulated with a membrane derived from the infected cell, which prevents antibody neutralization and allows them to escape the humoral immune response [12]. There have been similar challenges to developing an HCV vaccine due to multiple routes of transmission [13]. For example, HCV persists long after the initial infection, and in the presence of anti-HCV antibodies, this suggests that antibody-mediated protection may be resistant to cell–cell transmission [14]. The humoral immune response against HCV is also insufficient to protect against re-infection in many intravenous drug users and reinfection following liver transplantation [15,16]. HCV virus assembly and release are linked to the exosome secretory pathway [17]. Exosomes can transfer viral RNA to interferon-producing plasmacytoid dendritic cells [18]. The role of EVs and secretory vesicles in the transmission of viral infection and virulent factors is an emerging research area that could open novel therapeutic approaches for future pandemics [8,12,19,20].

The number of EVs in peripheral blood increases in COVID-19 patients, likely playing a critical role in coronavirus infection and pathogenesis [21]. SARS-CoV-2 RNA replication occurs in perinuclear double-membrane vesicles (DMVs), convoluted membranes (CMs), and tiny open double-membrane spherules (DMSs). These host-generated vesicles create a protective microenvironment for the replication of viral genomic RNA and the transcription of subgenomic mRNA (sgmRNAs) [22,23,24,25,26]. Commercially available vaccines are not very effective at preventing SARS-CoV-2 infection [27]. To address this limitation, we must understand the role of EVs in promoting cell-to-cell transmission, as well as the mechanisms of immune control and virus clearance. A significant barrier to addressing this limitation is the highly contagious and pathogenic nature of SARS-CoV-2 in humans, which restricts the study of active virus infection to biosafety level 3 (BSL-3) laboratories. This restriction hinders basic science research from contributing to the understanding of molecular virology and the development of drugs. The development of a BSL-2-compatible model system is needed to characterize the SARS-CoV-2 mode of transmission fully and to develop more effective vaccines.

**Figure 1:**
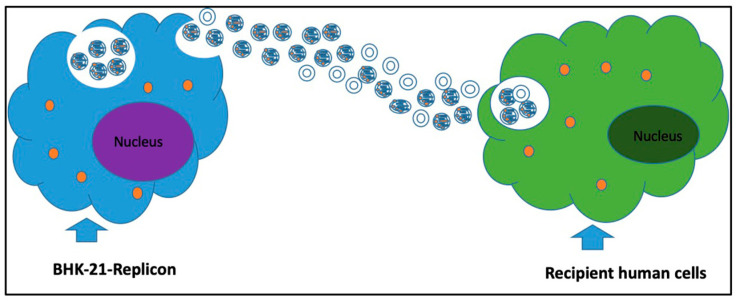
Diagram for the vesicle-mediated cell-to-cell transmission of SARS-CoV-2 RNA.

This limitation motivated us to examine the role of EV-mediated SARS-CoV-2 transmission using a replicon-based model system to exclude contributions from virus-dependent transmission. The primary aim was to test whether EV-mediated transfer of SARS-CoV-2 replicon RNA could establish a stable replication system in human cells. We hypothesized that EVs derived from BHK-21 could transfer replication-competent sub-genomic replicon SARS-CoV-2 RNA to develop a human cell replication model (Figure 1). Our results demonstrate that EV-mediated transmission of viral RNA is an efficient delivery system, enabling the generation of a productive viral replication system in alveolar lung epithelial cells and in gastrointestinal, neuronal, and endothelial cell culture models. This finding paves the way for a deeper understanding of the multifaceted pathogenic mechanism of SARS-CoV-2 mediated by EVs.

## 2. Materials and Methods

**Biocontainment:** The replicon cell culture studies were conducted following approval from the Tulane Institutional Biosafety Committee (IBC) and the Southeast Louisiana Veterans Health Care System (SLVHCS) Biosafety Committee. All experiments involving the SARS-CoV-2 replicon model were performed at BSL-2 tissue culture facilities housed at the SLVHCS and Tulane University Health Science Center. All personnel received BSL-2 training before participating in this study. The Centers for Disease Control (CDC) recommends using BSL-2 protocols to limit personal hazards, contamination, or accidental exposure while working with the SARS-CoV-2 replicon system. Personal protective equipment included BSL-2-compatible tissue culture hoods, lab coats, gloves, and eye/nose protection. Waste material was decontaminated with 10% bleach for 24 h before disposal.

**Reagents, inhibitors, drugs, antibodies, and antibiotics:** The commercial sources from which the chemicals and antibodies were purchased are as follows: imipramine hydrochloride (#15890), ketotifen fumarate (#20303), calpeptin (#14593), ritonavir (#13872), and nirmatrelvir (#35257) were obtained from Cayman Chemical Company (Ann Arbor, MI, USA); G-418 sulfate (#400-111P) was purchased from Gemini Bio-Products (West Sacramento, CA, USA). Actin (C-2) PE (#sc-8432), α-tubulin (TU-02) PE (#sc-8035), vimentin (V9) FITC (#sc-6260), and Anti-BP-PE (#sc-516141), as well as PE-labeled antibodies to Annexin A1, CD9, ARF6, asialo-glycoprotein receptor (ASGPR), and glypican-3, were obtained from Santa Cruz Biotechnology (Dallas, TX, USA). The SARS-CoV-2 viral nonstructural protein 1 (Nsp1) antibody (#PA5-116941) was purchased from Invitrogen (Carlsbad, CA, USA). Antibodies to β-actin and GAPDH were obtained from Santa Cruz Biotechnology (Dallas, TX, USA), antibodies to α-tubulin were purchased from Abcam (Cambridge, MA, USA), and HRP-linked anti-rabbit IgG (#7074S) and HRP-linked anti-mouse IgG (#7076S) antibodies were obtained from Cell Signaling Technology (Danvers, MA, USA).

**Cell culture:** Cell lines were cultured in DMEM supplemented with 1×-non-essential amino acids, 1× sodium pyruvate, 1× antibiotics, and 10% FBS at 37 °C and 5% CO_2_ in a humidified incubator. The following cell lines and commercial sources were used in this study: Human alveolar lung adenocarcinoma A549 cells (ATCC, CCL-185), Human airway epithelial cells, CALU-3 cells (ATCC, HTB-55) Human colorectal adenocarcinoma HT-29 cells (ATCC, HTB-38), Human colon adenocarcinoma, Caco2 cells (ATCC, HTB-37); human neuroblastoma SK-N-SH cells (ATCC; HTB-11), Human hepatocellular carcinoma HepG2 cells (ATCC; HB-8065), Human cardiomyocyte cell line Millipore AC-16 (EMD Millipore, SCC109), Mouse vascular endothelial cell line SVEC (ATCC), Human embryonic kidney cells HEK293T cells (ATCC; CRL-3216), Human kidney carcinoma rhabdoid tumor cell line (G401 ATCC; CRL-3020), all cell lines were purchase from ATCC (Manassas, VA, USA). Human hepatocellular carcinoma Huh-7.5 cells obtained from Charlie Rice Laboratory (Rockefeller University, New York city USA). Wild-type BHK-21 and stable SARS-CoV-2 replicon cell clones (clone 1–8) in BHK-21 cells were obtained from our collaborator Tony Wang’s laboratory (Silver Spring, MD, USA) [28].

**Extracellular vesicle isolation, and quantitation:** SARS-CoV-2 sub-genomic replicon cell line was developed in the baby hamster kidney fibroblast cell line BHK-21(CCL-10) (clone 8) and cultured in Dulbecco’s modified Eagle Medium supplemented with 5% penicillin, 10% fetal calf serum, sodium pyruvate and non-essential amino acids at 37 °C with 5% carbon dioxide and 200 microgram/mL of G-418 (Sigma, St. Louis, MO, USA). The sub-genomic replicon system was prepared by replacing the S gene with a nano luciferase gene and the M and E genes with the neomycin phosphotransferase (Neo) gene. The replicon plasmid sequence was confirmed by both restriction digestion analysis and DNA sequencing [27]. Cells were cultured with regular medium changes in three-day intervals. EVs were isolated from cell-free supernatants of BHK-21 replicon cells using a previously published protocol [29,30]. Briefly, cellular debris was removed from freshly harvested cells by centrifugation at 2000× *g* for 30 min. Cleared supernatants were transferred to a new tube and mixed with one-third volume of a 50% stock solution of PEG 3350 (Amazon, Seattle, WA, USA) plus 3.75 M NaCl (Sigma-Aldrich, St. Louis, MO, USA). Samples were mixed gently by inverting the tubes three times, and the EV was precipitated overnight. The following day, samples were centrifuged at 10,000× *g* for one hour at 4 °C. The resulting supernatant was carefully removed, and the EV pellet was resuspended in 1 mL of PBS. To remove PEG from the EV preparation, the entire 1mL of solution was passed through a G-200 column. The EV preparations were stored at −20 °C for short-term use or at −80 °C for long-term storage.

**Nanoparticle tracking analysis (NTA):** EV concentration and size distribution were determined using NanoSight (Model NS300-NTA3300, Malvern Panalytical, Worcestershire, UK) equipped with a 532 nm green laser and a 565 nm long-pass filter for fluorescence detection. Technical triplicate measurements in light scattered mode (LSM) were performed for each sample used in this study. The Brownian motion of EVs was visualized in real time, while liquid-state light scattering was used to measure EV concentration and size. A syringe pump allows particles to move freely within the tube. EVs in suspension are passed through a flow chamber and illuminated by a laser source to analyze the total particle concentration using NTA software (v3.2, Malvern Panalytical, Worcestershire, UK), expressed as particles/mL. Initially, LSM measurements were performed to analyze EV concentration using a 30 s recording per individual replicate. A washing step was performed between each measurement using ultrapure particle-free water. The NTA instrument has a limited dynamic range for particle concentration (10^4^–10^8^ particles/mL). Therefore, all samples were diluted to a maximum concentration of 1 × 10^8^ particles/mL to obtain accurate concentration measurements within the instrument’s detectable range.

**Detection of exosomal and microvesicle markers in the replicon EV by Fluorescence-NTA (F-NTA):** Phycoerythrin (PE) -conjugated anti-human CD9, anti-human ARF6, and anti-human annexin A1, PE-labeled glypican-3, and PE-labeled ASGPR were purchased from Santa Cruz Biotechnology (Dallas, TX, USA). Extracellular vesicles were isolated from replicon by precipitation using a kit (Invitrogen) or PEG precipitation. One hundred microliters of EV samples were incubated overnight at 4 °C with shaking in the presence of PE-labeled antibodies (1:50 dilution). The next day, EVs were purified by Sephadex G-200 size exclusion chromatography. PE-antibody-labeled EV samples were diluted 1:10 and immediately processed for F-NTA analysis.

**Cryo-TEM for exosome characterization**: Transmission electron microscopy (TEM) and Cryo-TEM analyses were performed on EVs purified from BHK-21 replicon cell culture supernatants after overnight PEG precipitation. The exosome pellet was resuspended in PBS. Cryo-TEM was conducted to demonstrate the purity and size of exosomes released from the BHK-21 replicon culture using an FEI G2 F30 Tecnai TEM operating at 150 kV, located on the uptown Tulane University campus.

### 2.1. EV Delivery Efficacy

EV transduction efficiency was determined by labeling with a fluorescent dye. In brief, 100 µL EV sample (1 × 10^10^ particles/mL in PBS (pH 7.2) was incubated with 1 µL dye (Vybrant™ DiD Cell-Labeling Solution, V22887; Thermo Fisher Scientific, Waltham, MA, USA) for 30 min at room temperature in the dark using a standard protocol [29,31]. Fluorescence-stained EVs were diluted to 1mL in PBS. Free DiD dye was separated from the labeled EV preparation by Sephadex G-200 column purification. The column-purified DID-labeled EV samples were used for uptake studies. Cells were cultured in T25 tissue culture flasks in DMEM with 10% FBS, 100 IU/mL penicillin, streptomycin, glutamine, and non-essential amino acids until they reached 75% confluency. For EV uptake studies, 1 × 10^8^ particles of the sterile, EV-enriched solution were added directly to the growth medium overnight (12 h). The next day, the medium was removed and washed with 10 mL of PBS. The cells were then examined under fluorescence microscopy (EVOS System, Invitrogen) and imaged at 40× magnification. The mean fluorescence intensity (MFI) was measured using ImageJ (version 1.54g, National Institutes of Health, Bethesda, MD, USA) for all samples relative to untreated A549 cells.

### 2.2. EV-Mediated Delivery of Viral RNA

Supernatants from replicon cells (12 mL) were centrifuged at 2000 rpm for 10 min to remove cell debris, then split into three sterile 15 mL tubes. In one tube, EV depletion from the replicon supernatants was performed by PEG precipitation. In the second tube, we depleted RNA/RNPs/EVs by passing the 3 mL supernatants through a DEAE-Sephadex column (Sigma, catalog no. 047K1595). Another 3 mL supernatant collected in the third tube was passed through an RNA purification column (Invitrogen). The PureLink spin column kits are a family of fast, simple, and cost-effective nucleic acid purification kits for preparing genomic DNA, plasmid DNA, total RNA, and microRNAs. RNA binds to this glass-fiber filter technology using a syringe filter. All three samples were incubated with wild-type BHK-21 cells for six hours at 37 °C in a tissue culture incubator. After this step, the inoculum was removed, and the cells were washed once with 10 mL of complete growth medium. They were then incubated with growth medium supplemented with G-418 (200 micrograms/mL). After 4 weeks, the media were removed, the cells were washed with PBS, and the cells were fixed in 5 mL of methanol for 15 min. After this step, cells were stained with Giemsa dye (Sigma) using a standard protocol for overnight. The next day, the dye was removed, and the plates were washed with distilled water.

**Generation of stable replicon system in human cells by EV transfer:** Cell lines were cultured in a T75 flask in a growth medium DMEM supplemented with 1×-non-essential amino acids, 1× sodium pyruvate, 1× antibiotics, and 10% FBS at 37 °C and 5% CO_2_ in a humidified incubator at the SLVHCS. Cells were selected for the EV transfer experiment when they reached 50% confluency. Cell culture supernatant (10 mL) was centrifuged at 2000 RPM for 10 min to remove cell debris. Afterward, cell-free supernatants were carefully removed to a new 15 mL tube. EV purification was carried out by passing through Sephadex G-200 column in the tissue culture hood. The flow through liquid, mainly the EV, was incubated with human cells overnight at 37 °C. The next day, the EV inoculum was removed and washed with PBS. Cells were cultured with growth media containing 20 μg/mL G-418 to select cell clones that replicated SARS-CoV-2 RNA. Cells were maintained in culture until they formed viable colonies. Individual cell colonies were picked and expanded with stable cell lines prepared in closed-neck T75 flasks. The success of SARS-CoV-2 RNA replication was measured by luciferase assay.

### 2.3. Immunostaining

Culture cells were immobilized onto glass slides either by cytospin or direct culture on coverslips. Before immunostaining, cells were fixed in ice-cold acetone for 30 min, incubated with a blocking solution (BioCare Medical) for 10 min, and then incubated with a primary antibody (1:200 dilution) overnight. After the primary antibody incubation, slides were washed 3 times in Tris-buffered saline (TBS) (pH 8.0), and incubated with a MACH 4 mouse probe (BioCare Medical, UP534) for 10 min. MACH 4 HRP Polymer (BioCare Medical, MRH534) for 30 min each, then washed 3 times using TBS. Finally, cells were treated with chromogen (Biocare) for 1–5 min. The slides were then counterstained with hematoxylin for 30 s and Tacha’s Bluing Solution (BioCare Medical, HTBLU) for 30 s, dehydrated with 95% and 100% alcohol, mounted, and observed under light microscopy (EVOS System, Invitrogen). Photographs were taken at 40× magnification.

**Western blotting:** Cultured cells were pelleted by centrifugation at 1000 rpm for 5 min. The cell pellet was washed in PBS and suspended in lysis buffer. Cells were homogenized by passing through a syringe with a 27-gauge needle at least 10 times. Approximately 200 µg of protein was loaded per lane to ensure an optimal signal on Western blotting. Before loading, the lysates were mixed with 5 µL of blue SDS sample loading buffer and 2.5 µL of sample reducing agent. The entire mix was incubated in a heater at 80–100 °C for 5 min. Samples were loaded into a 4–20% SDS-PAGE gel. A pre-stained molecular size marker was loaded to verify protein size. The samples were run for one hour at 142 V until they reached a target threshold at the bottom of the gel. Nylon membranes were pre-soaked in transfer buffer, and proteins were transferred by running at 52 V for 30 min. The membrane was carefully removed, and a cut was made at the left corner to determine the orientation of your samples. The membrane was then incubated in a blocking reagent consisting of 0.5 g of dry non-fat milk dissolved in 10 mL of Tris-buffered saline solution containing 0.1% Tween 20 (TBST) for 1 h at room temperature (RT). After the membrane had been washed twice in TBST solution for 5 min each, the membrane was incubated overnight at 4 °C with anti-Nsp1 antibody diluted in TBST at a concentration of 1:1000. Beta-actin antibody (Santa Cruz) was diluted at 1:000 and tubulin antibody (Abcam) was diluted at 1:1000. GAPDH antibody (Santa Cruz) was diluted at 1:1000. Then, the membrane was washed three times in TBST for 5 min each at RT. Then, the washed membrane was incubated with the secondary antibody (mouse anti-rabbit) diluted 1:1000 in TBST at RT for 1 h. After the membrane has been washed in TBST three times for 5 min each, the membrane was developed using an ECL detection kit, and Nsp1 detection was performed using a BIO-RAD phospho-imaging system (Pathology, Room No. 6710). The detection of GAPDH as an internal control was performed using the same membrane. First, the membrane was stripped in 10 mL of 0.25 mM NaOH for 1 h, then washed 3 times in TBST for 5 min each at RT. Then, the membrane was blocked in 10 mL of TBST containing 0.5 g non-fat dry milk for one hour at RT. Then, the membrane was washed twice in TBST for 5 min each at RT and incubated overnight at 4 °C with an anti-GAPDH antibody diluted in TBST at 1:1000, after washing three times for 5 min each. Following incubation with the secondary antibody (Rabbit anti-mouse antibody) for 1 h at RT, the membrane was washed 3 times in TBST for 5 min each at RT. The development of the membrane and the detection of the GAPDH signal were performed as described above.

### 2.4. Antiviral Treatment and Luciferase Assays

Luciferase assays were performed according to the manufacturer’s instructions (Promega medicine, Nano-glo ^®^ Luciferase assay REF N110, USA). Briefly, replicon cells (1 × 10^5^) were cultured in 12-well plates. After 24 h, the culture media were removed, and fresh media without G-418 were added, along with various concentrations of antivirals or EV inhibitors. The treatment was continued for 72 h. Afterward, cell lysates were prepared, and relative light units (RLUs) were measured using Lumat LB 9507 (Berthold, Germany). Luciferase activity was normalized to protein concentration. The percentage of reduction in luciferase activity was compared with that of the untreated control.

**Statistical analysis:** All antiviral treatment and luciferase measurement experiments were carried out in triplicate. All results were expressed as mean + SE (standard error) and n = 3. A comparison between two groups was performed with a Student’s *t*-test. The *p*-value for the Student’s *t*-test was significant when *p* < 0.05

## 3. Results

**Figure 2:**
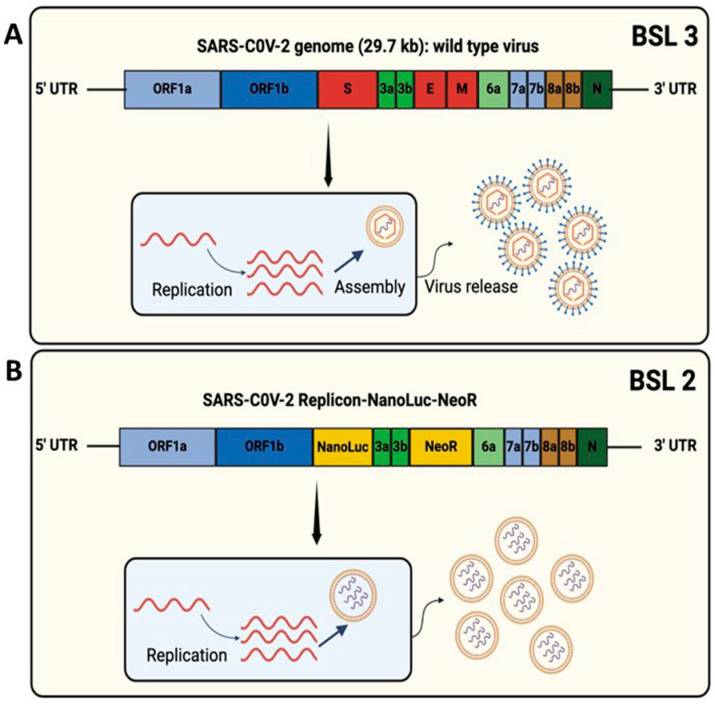
Diagram showing the difference in biosafety levels between the cell culture model involving whole SARS-CoV-2 genomic RNA and sub-genomic replicon RNA (SARS-CoV-2-Rep-NanoLuc-Neo). (**A**). Top: genome organization of the SARS-CoV-2 wild-type virus that requires the use of biosafety level 3 (BSL-3) containment. Work on this model needed a BSL-3 facility. (**B**). Bottom, the organization of sub-genomic replicon RNA (SARS-CoV-2-Rep-NanoLuc-Neo) with deletion of the spike (S), envelop (E), and membrane (M) genes. This replicon RNA autonomously replicates intracellularly without producing infectious virus particles. This model does not produce infectious virus particles. Only viral RNA is packed in extracellular vesicles (EVs). Work on this model needed a biosafety level 2 facility.

**SARS-CoV-2 sub-genomic RNA replication promotes EV release in BHK-21 cells:** A full-length SARS-CoV-2 infectious clone developed using an in vitro ligation approach replicates efficiently, similar to clinical virus strains. However, research in infectious full-length SARS-CoV-2 cell culture models requires BSL-3 containment due to the virus’s highly contagious and virulent nature and the risk of transmission to humans (Figure 2A). Two prior publications have attempted to address the role of EV as a carrier of viral RNA or the whole virus [32,33]. They have shown that SARS-CoV-2 RNA and virus particles are present in extracellular vesicles from COVID-19 patients, and these EVs could transfer viral RNA into cell culture. The similar size of EVs and viral particles makes it difficult to distinguish the role of the canonical virus particle from EV-mediated SARS-CoV-2 RNA transmission. To avoid this confusion, we used a stable cell culture model harboring sub-genomic SARS-CoV-2 RNA that does not produce virus particles. Among multiple BHK-21 clones tested, clone eight was selected for EV transfer experiments because it supports very high-level replication of the sub-genomic replicon RNA (SARS-CoV-2-RepNanoLuc-Neo), as described previously [28]. This sub-genomic clone replaced the spike gene (S) with the nano luciferase (Nano Luc) reporter gene. The absence of S, E, and M genes in the replicon clones has been confirmed previously. The envelope (E) and membrane (M) genes were replaced with the neomycin phosphotransferase gene, enabling stable culture of replicating cells in growth media containing the G-418 selection antibiotic. This model does not generate complete virus particles. In replicon cells, the S, M, and E genes are replaced with those encoding NanoLuc and NeoR. The gene encoding ORF6 is lost because its transcriptional regulatory sequence body (TRS-B) resides in the M gene, which is also deleted in the replicon RNA. Hence, cells harboring the replicon would express ORF1a/b mRNA, which produces nonstructural proteins, and six coding mRNAs, namely, Nanoluc, NeoR, ORF3a, ORF7a, ORF8, and nucleoprotein (NP). The temporal expression of these small RNAs has been confirmed in replicon cells by the Wang laboratory [28]. It is expected that the replicon cells will generate SARS-CoV-2 viral RNA, packaged with nucleoprotein, double-stranded RNAs, replicative intermediates, and replicon RNA, in membrane-derived extracellular vesicles (Figure 2B).

**Figure 3:**
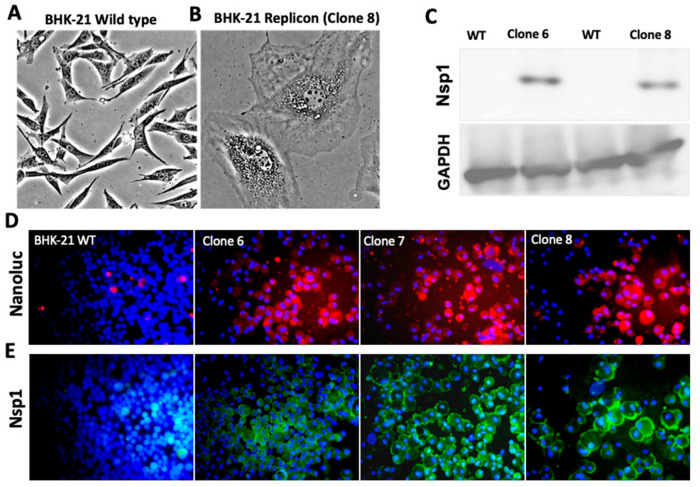
Cell morphological features of SARS-CoV-2 sub-genomic replicon cells in BHK-21 cells. (**A**,**B**). Light microscopic view of the morphology of wild-type BHK-21 cells (40×) and BHK-21 replicon (40×). We identified several prominent morphological changes associated with SARS-CoV-2 autonomous replicon RNA replication, including large size, multiple shapes, accumulation of double-membrane vesicles (DMVs), syncytia formation with cell–cell fusion, and cytoplasmic protrusions. (**C**). Western blot showing expression of viral Nsp1 (19 kDa) and GAPDH (37 kDa) in the lysate prepared from wild-type BHK-21 and SARS-CoV-2 replicon cells. (**D**). Expression of Nano Luc protein between wild type and BHK-21 replicon by immunofluorescence (40×). Rounded cells with intense staining of Nano Luc protein in different SARS-CoV-2 sub-genomic BHK-21 replicon clones. (**E**). Expression of viral protein Nsp1 between wild-type BHK-21 and replicon by immunofluorescence staining (40×). Most of the cells show cytoplasmic expression of Nsp1. Photographs were taken at 40× magnification.

Replication of positive-stranded RNA viruses occurs in the cytoplasm. It is known to remodel the host cell membrane, thereby hiding the genomic RNA from the host’s innate antiviral response pathways. SARS-CoV-2 replication induces dramatic changes to the actin cytoskeleton, resulting in morphological changes [34]. We compared the profiles of wild-type and BHK-21 replicon cells under light microscopy to determine whether SARS-CoV-2 sub-genomic RNA replication altered BHK-21 cell morphology. Consistent with other infectious coronavirus cell culture models, BHK-21 replicon cells exhibited an enlarged morphology, characterized by the accumulation of numerous DMVs around the nucleus and cell–cell fusion, leading to syncytia formation (Figure 3A,B). This observation is consistent with DMV structures observed in cells infected with SARS-CoV-2 [31]. SARS-CoV-2 sub-genomic RNA replication has been confirmed by detecting Nsp1 by Western blotting in BHK-21 cells with or without SARS-CoV-2 RNA replication (Figure 3C, full blot included in Supplementary Figure S1). BHK-21 replicon cells also exhibited high-level viral protein expression after staining with antibodies targeting the NanoLuc protein (Figure 3D) or the viral Nsp1 protein by immunofluorescence staining (Figure 3E). These results indicate that the sub-genomic replicon system in BHK-21 cells is stably replicating SARS-CoV-2 sub-genomic RNA. The cellular machinery used for EV production and secretion is also employed during viral RNA replication; therefore, viral replication increases EV production [35].

**Figure 4:**
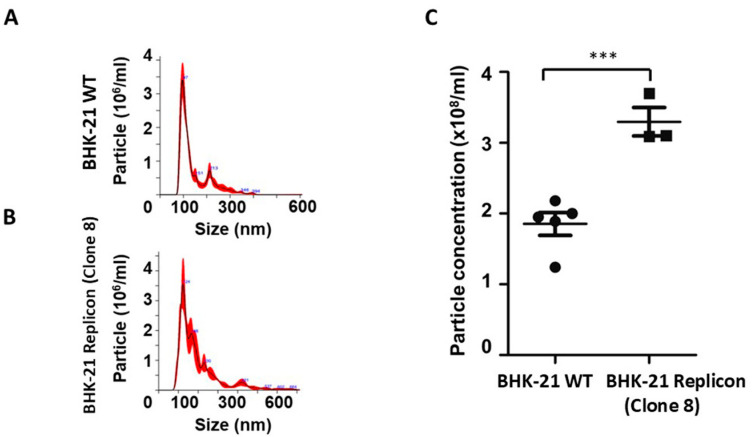

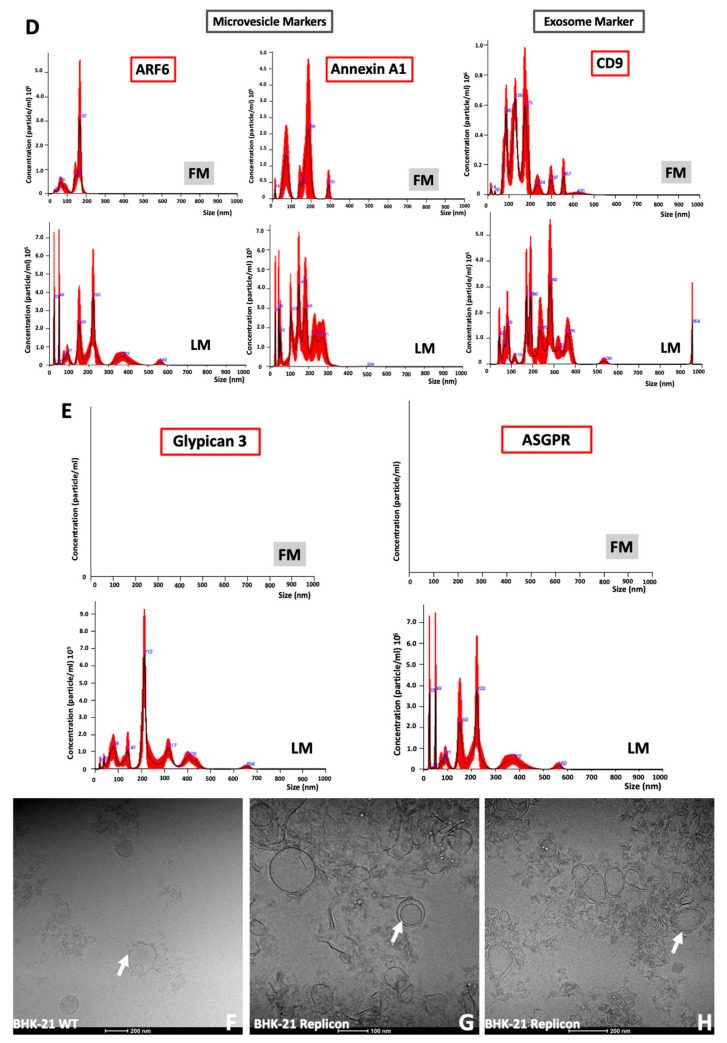
SARS-CoV-2 replicon cells produce more extracellular vesicles. Extracellular vesicles were isolated from cell culture supernatants after precipitation. The EV size between wild-type BHK-21 and the replicon clone was determined by nano tracking analysis. (**A**,**B**). The size of EVs isolated from wild-type and replicon cells. (**C**). The concentration of EVs produced by wild BHK-21 cells and BHK-21 replicon. (**D**). F-NTA quantification data showing the population diversity of EVs produced in replicon culture. (**E**). F-NTA analysis of EVs stained with two control, unrelated PE-labeled antibodies to glypican-3 and asialoglycoprotein receptor (ASGPR). (**F**–**H**). Transmission electron microscopy of EVs isolated from wild-type (**F**) and replicon cells (**G**,**H**). Arrows refer to extracellular vesicles. *** *p*-value < 0.0001, Student’s *t*-test was used.

Changes in EV production caused by viral RNA replication were examined in cell-free supernatants following PEG precipitation, and NTA was used to quantify their concentration. NTA revealed EVs derived from BHK-21 replicon were heterogeneous in size, comparable to wild-type BHK-21 control cells (Figure 4A,B). However, the quantity of EV release from the BHK-21 cells was significantly higher (*p* > 0.001) in the replicon line than in control cells (Figure 4C). EVs are generally classified into two major subtypes: (i) exosomes: small vesicles (30–150 nm in diameter); and (ii) microvesicles (MVs): vesicles ranging from 100 to 1000 nm in diameter, formed by the outward budding of the plasma membrane. We tested the immunoreactivity of EV generated from stable replicon culture after column purification. The diversity of EVs produced in the replicon culture was confirmed using PE-conjugated antibodies specific to microvesicles (ARF6, annexin A1) and exosomes (CD9) (Figure 4D). To verify the specificity of fluorescence NTA-antibody staining of EVs, the experiment was repeated with two unrelated control PE-labeled antibodies targeting human glypican-3 and asialoglycoprotein receptor (ASGPR), both of which are available in the laboratory. We show that the fluorescence peak was not detected when stained with the two control antibodies (Figure 4E). Using fluorescence-labeled synthetic beads, we previously determined the sensitivity of F-NTA is 10^4^–10^8^ per/mL [29]. EV staining with fluorescence-labeled unrelated antibody signal remained below 10^4^ particles /mL, whereas EV staining with annexin A1, ARF7, and CD-9 was in the range of 10^5^ to 10^6^ particles/mL. The total number of EVs shown in the bottom panel was comparable among all samples.

Transmission electron microscopy (TEM) analysis was performed to visualize the EVs’ morphology, given its nanometer resolution. To observe their native, spherical morphology without artifacts, cryo-electron microscopy (cryo-EM) is the preferred method, as it involves plunge-freezing the sample without chemical fixation or staining. Extracellular vesicles are a heterogeneous group of nanoparticles (30–1000 nm in diameter) secreted by cells with a single phospholipid bilayer. TEM images capture the distinct morphology of these vesicles, with DMVs appearing as concentric double-bilayer structures, whereas typical EVs exhibit a single membrane. DMVs are intracellular compartments typically induced by positive-strand RNA viruses (such as coronaviruses) to serve as platforms for viral replication. TEM analysis shows that DMVs are clearly identified by the presence of two distinct, closely opposed membrane bilayers surrounding the lumen (Figure 4G,H). All these data support the hypothesis that SARS-CoV-2 replicon culture sheds single- and double-membrane vesicles of heterogeneous sizes.

**Figure 5:**
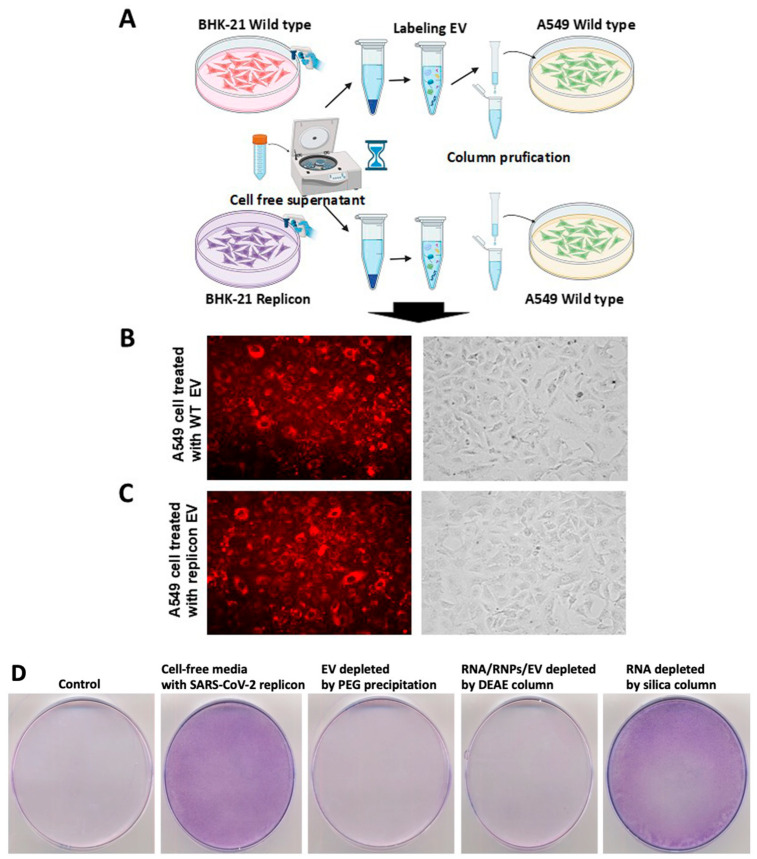
Experimental setup for EV uptake. (**A**). The overall experimental plan for EV purification, labeling with fluorescence dye (DiD), column purification of labeled EV, and uptake studies in human A549 lung epithelial cells in culture. EVs were purified from wild-type BHK-21 and replicon cells by precipitation. Labeled EVs were separated from free dye by Sephadex G-200 column purification. Labeled EVs from wild-type and replicon cells were incubated with freshly cultured A549 cells for 12 h. The difference in uptake efficiencies was assessed under a fluorescence microscope. (**B**). Uptake efficiency of EVs derived from wild-type BHK-21 cells. (**C**). Uptake efficiency of EVs derived from BHK-21-SARS-CoV-2 replicon cells. Photographs were taken at 40× magnification. (**D**). The EV-dependent uptake of SARS-CoV-2 RNA in wild-type BHK-21 cells. BHK-21 cells were incubated with EV-depleted media, RNA/RNPs/EV-depleted media, and viral RNA-depleted media for 6 h. BHK-21 cells without any treatment were used as a control for G-418 selection. BHK-21 cells treated with replicon media without any treatment were used as a positive control. Then, the cells were cultured in growth medium containing G418 (200 μg/mL). After four weeks, the cells were stained with Giemsa. Cells incubated with RNA-free media continue to grow, whereas cells incubated with EV-depleted culture media do not.

**EV transduction efficiency and its direct role in the transfer of SARS-CoV-2 RNA to naïve cells:** To test the virion-independent and extracellular vesicle-dependent transmission of SARS-CoV-2 RNA to human cells, we first assessed EV transduction efficiency in cultured cells using a fluorescent dye. EV-mediated cell–cell transfer was investigated using BHK-21 replicon cells as donors and A549 as target cells. A standard precipitation protocol was used to isolate EVs from wild-type and BHK-21 replicon cells (Invitrogen). EVs were labeled with a lipophilic membrane fluorescent dye (DiD, V22887, Thermo Scientific) according to the manufacturer’s protocol [29,30]. To separate the free unlabeled DiD dye molecules from the labeled vesicles, BHK-21 replicon EVs were first labeled with the fluorescent lipophilic dye DiD and then purified using a Sephadex G-200 column as outlined in Figure 5A. The uptake of DiD-labeled EVs from BHK-21 wild-type and BHK-21 SARS-CoV-2 replicon was then calculated after incubation with A549 cells (Figure 5B,C). Cytoplasmic fluorescence from the DiD-labeled EVs was detectable throughout the culture 24 h after exposure, indicating that EVs are a highly efficient delivery system. EVs produced by wild-type BHK-21 and replicon cells were equally effective in delivering the lipophilic dye cargo.

A second set of control experiments was performed to investigate the contribution of free viral RNA (RNA), nucleocapsid-bound RNA (RNP), and extracellular vesicles (EVs) in mediating SARS-CoV-2 replicon transmission. We have performed three control experiments to verify the role of EV-mediated transmission of viral RNA (Figure 5D). The first control experiment was conducted by depleting EVs from the replicon supernatants via PEG precipitation. We found that when wild-type BHK-21 cells were incubated with EV-depleted media, they did not grow in media supplemented with G-418. The second experiment was performed by depleting RNA/RNPs/EVs from the replicon culture supernatant using DEAE-dextran column purification [36,37]. We presented data indicating that when wild-type BHK-21 cells were incubated with RNA/RNPs/EV-depleted culture media, they did not grow in culture media supplemented with G418. The third control experiment was performed by depleting RNA from the culture supernatants by passing them through an RNA purification column (Invitrogen) [38]. We show data indicating that when BHK-21 cells were cultured with RNA-depleted media, they survived G-418 treatment and continued to grow (Figure 5D). Wild-type BHK-21 cells without replicon-containing media did not grow in the presence of G-418, whereas wild-type BHK-21 cells cultured with replicon media survived G-418 selection and formed cell colonies. These three control sets of data support our conclusion that SARS-CoV-2 transfer is primarily mediated by EV rather than by free RNA circulating in the media.

**Figure 6:**
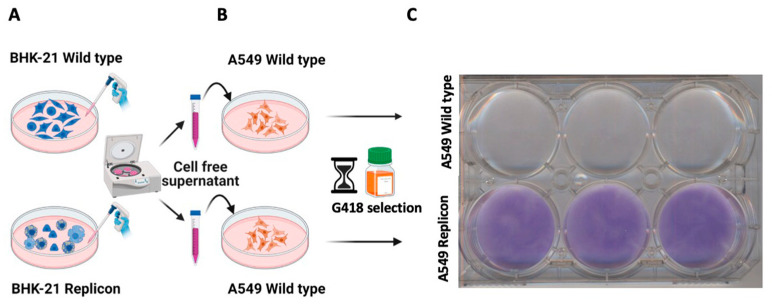
Experimental setup to test extracellular vesicles-mediated transfer of SARS-CoV-2 replicon RNA to naïve mammalian cells. (**A**) The overall experimental plan for the purification of EVs from wild-type BHK-21 and BHK-21 replicon cells by precipitation and centrifugation, as well as uptake studies in A549 cells. (**B**) The specific uptake of SARS-CoV-2 RNA by A549 cells was determined. (**C**) Upper panel. Naïve A549 cells incubated with EVs isolated from wild-type BHK-21 did not survive G-418 selection. The lower panel. Naïve A549 cells incubated with replicon EV survived and formed G-418-resistant cell colonies at day 7, indicating EV-mediated transfer of SARS-CoV-2 replicon RNA.

**BHK-21 replicon-derived EVs transfer SARS-CoV-2 replicon RNA and induce G-418 antibiotic resistance in human A549 cells:** In the new set of experiments, these observations of a functional payload and virus-independent EV transfer were extended, and delivery efficiency experiments were repeated using SARS-CoV-2 RNA from replicon cells in human A549 cells (Figure 6A). First, cell-free supernatants were prepared from wild-type and replicon BHK-21 cells by centrifugation at 10,000× *g* for 30 min, and the supernatants were carefully collected in a new tube and incubated overnight with cultured naïve human lung alveolar epithelial cells (A549) (Figure 6B). The next day, the cells were washed three times and then incubated in growth media supplemented with G-418 (20 μg/mL). Since SARS-CoV-2 replication in human cells induces cellular toxicity, we decreased the concentration of G-418 to 20 micrograms/mL. EVs derived from mock-transfected, wild-type BHK-21 cells could not confer resistance to the A549 cells and therefore remained sensitive to G-418 and served as the negative control (upper panel Figure 6C). In contrast, A549 cells incubated with EV derived from a BHK-21 replicon culture developed colonies resistant to G-418-mediated cell killing (lower panel, Figure 6C), supporting the successful transmission of the replicon RNA to A549 cells.

**Figure 7:**
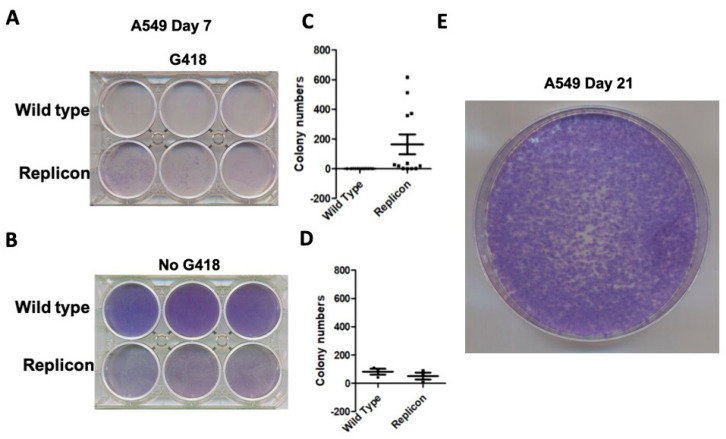
Experimental plan for selection of SARS-CoV-2 replicating cells using low-dose G-418 (20 microgram/mL) for the development of stable A549 replicon cells. (**A**,**C**) G-418 selection increased the number of replicon-containing A549 cell colonies. (**B**,**D**) Without G-418 selection, SARS-CoV-2 replicating A540 cells show reduced cell proliferation as compared to A549 cells that received EV from wild-type BHK-21 cells. (**E**) Stable selection of A549 cells replicating SARS-CoV-2 sub-genomic RNA.

Giemsa colony staining demonstrated marked proliferation of A549 cells receiving replicon EVs, compared with cell death in cells receiving wild-type EVs (Figure 7A). Colony formation with and without G-418 was quantitatively confirmed by counting the number of Giemsa-stained colonies (Figure 7C). In the absence of G-418 treatment, A459 treated with wild-type EVs proliferated at a faster rate than cells receiving replicon EVs (Figure 7B–D). A549 cells treated with replicon EVs continued to proliferate, spawn new colonies, and remained stable in culture in the presence of G-418 (Figure 7E).

**Figure 8:**
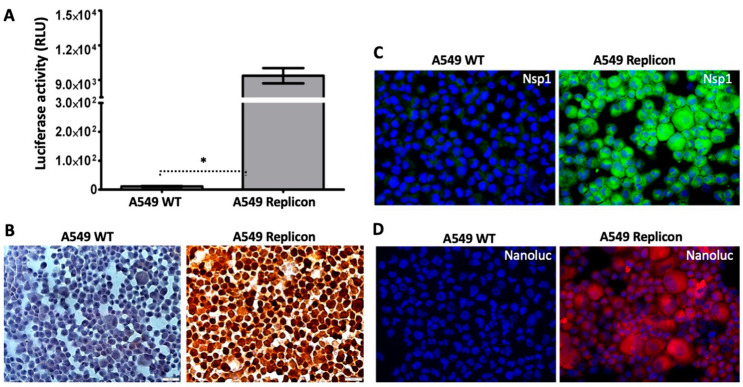
Verification of SARS-CoV-2-Rep-NanoLuc-Neo replicon RNA in A549 cells. (**A**). Nano luciferase expression in A549 replicon cells was measured after one month. * *p*-value < 0.001, Student’s *t*-test was used. Luciferases were measured three times for each scale bar (n = 3). (**B**). Expression of Nsp1 protein in wild-type A549 cells and A549 replicon cells by immunocytochemical staining of culture cells. (**C**). Expression of Nsp1 by immunofluorescence staining between wild-type and replicon-replicating A549 cells. (**D**). Nano luciferase protein expression by immunofluorescence staining of wild-type and A549 replicon cells in culture. Photographs were taken at 40× magnification.

**Verification of sub-genomic replicon replication in A549 cells:** Nano luciferase expression driven by the SARS-CoV-2 replicon RNA was used to validate replication in A549 cells. Nano luciferase activity for the wild type and A549 replicon was normalized to protein concentration, which demonstrated increased expression in A549 replicon cells (Figure 8A). Protein level expression of viral Nsp1 was detected in the cytoplasm of the A549 replicon cells, but was absent in the wild-type A549 cells (Figure 8B). Immunofluorescence staining for nano luciferase and Nsp1 confirmed abundant expression throughout the A549 replicon monolayer, with no immunofluorescence signal detected in the wild type (Figure 8C,D).

**Figure 9:**
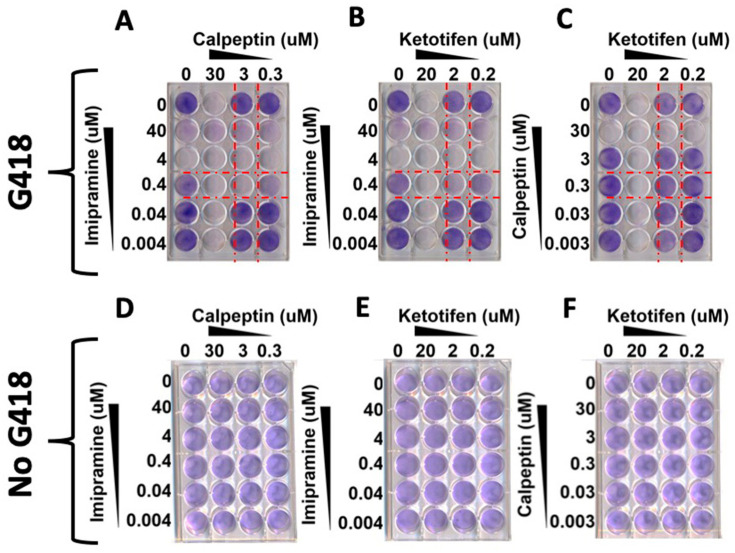
Analysis of EV inhibitors combination treatment using the SARS-CoV-2 replicon system in A549 cells. The upper panel shows the antiviral efficacy based on replicon cell killing in the presence of G-418. (**A**–**C**): show antiviral potency of EV inhibitors in combination treatment (**A**,**B**). The lowest concentration of imipramine required for 100% inhibition of SARS-CoV-2 replication (no colonies) was reduced by 10-fold when combined with calpeptin or ketotifen in vitro. (**C**). The lowest concentration required to inhibit calpeptin or ketotifen, necessary for 100% inhibition of SAR-CoV-2 replication, was not reduced when these two drugs were combined. The following formula was used to quantify the interaction between two compounds. A/MIC A + B/MIC B = FIC A + FIC B = FIC index. (**D**–**F**). Cellular cytotoxicity of combination drug treatment assessed by Giemsa staining, indicating no cellular cytotoxicity.

Since SARS-CoV-2 RNA replicates in DMV, we tested whether SARS-CoV-2 replication is sensitive to EV inhibitors. Three EV inhibitors were selected: imipramine, known to inhibit EV formation and release; ketotifen, which blocks EV release; and calpeptin, which blocks calcium influx, an upstream inhibitor of EV release [39]. Among these, imipramine is an FDA-approved tertiary amine tricyclic antidepressant that prevents translocation of acid sphingomyelinase (aSMase), therefore inhibiting extracellular vesicle formation [26]. Ketotifen is an antihistamine medication that inhibits the release of vasoactive substances from mast cells and is an intracellular calcium inhibitor that also reduces EV secretion. Calpeptin is a cysteine protease inhibitor that also blocks calcium influx, therefore inhibiting EV release. A549 cells were cultured with different exosome inhibitors for 48 h, and the expression of Nsp1 was measured by fluorescence microscopy (Supplementary Figure S2). Robust Nsp1 expression was detected in the positive control A549 replicon. EV inhibitors, including imipramine, inhibited replication as evidenced by Nsp1 suppression with calpeptin, yielding partial inhibition. In contrast, ketotifen had a minimal effect on Nsp1 expression compared to the positive control. Sustained antiviral effects of EV inhibitors were confirmed by a decrease in the number of Giemsa-stained G-418-resistant cell colonies (Figure 9). Imipramine showed synergistic antiviral activity with calpeptin and ketotifen (F < 0.1) (Figure 9A,B), whereas calpeptin in combination with ketotifen was not synergistic (F > 1) (Figure 9C). A549 replicon cells in culture treated with similar concentrations of EV inhibitors without G-418 were not toxic to the cells (Figure 9D–F).

**Figure 10:**
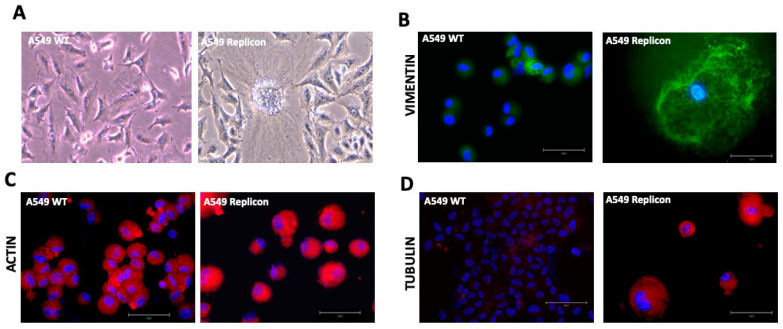
SARS-CoV-2 sub-genomic RNA replication induces cytoskeletal architecture remodeling. (**A**). Light microscopy visualization of cell shape changes and cytoskeletal abnormalities in A549 replicon cells not in the wild type A549 cells. (**B**). Immunofluorescence staining showing the autonomous replication of sub-genomic SARS-CoV-2 RNA in A549 cells induced expression of vimentin. (**C**). Actin expression is induced in A549 cells replicating SARS-CoV-2 sub-genomic RNA. (**D**). Fluorescence staining shows that the autonomous replication of SARS-CoV-2 in A549 cells induces the expression of alpha-tubulin. The expression of cytoskeletal proteins (actin, vimentin, and tubulin) increased in A549 cells replicating SARS-CoV-2 RNA. Photographs were taken at 40× magnification.

**SARS-CoV-2 replication induces cytoskeletal remodeling in A549 cells:** Viral replication alters the expression of actin filaments (AFs), microtubules (MTs), and intermediate filaments (IFs), leading to morphological changes in the infected cell [34]. AFs are involved in the movement of submembrane regions during cell–cell contact, vesicular spread, and shape change. Actin forms monomeric and spherical forms called globular actin (G-actin). AFs form shorter protrusions, such as the development of lamellipodia, membrane ruffles, filopodia, microvilli, or podosomes (looser bundles). They are organized in parallel stress fibers (tightly packed bundles) [34]. Actin filaments also form tubular F-actin-rich structures called TNTs (tunneling nanotubes). They connect the cytoplasm of neighboring and distant cells by mediating efficient intercellular communication, thereby maintaining homeostasis in cellular physiological processes. MTs are long, hollow, cylindrical polar structures with dynamic plus-end and minus-end, assembled by heterodimers of α- and β-tubulin. MTs are involved in the virus replication complex in the perinuclear zone. Intermediate filaments are of six types, including keratins (types I and II); desmin, glial fibrillary acidic protein (GFAP), and vimentin (type III); neurofilaments, nestin, and α-internexin (type IV); nuclear lamins (type V); and filensin and phakinin (type VI or others) [40]. IFs are more stable, usually surround the nucleus, and extend throughout the cytoplasm, serving as scaffolds and participating in intracellular organization, membrane trafficking, and signal transduction. Abnormalities in IFs lead to severe pathogenesis, including epithelial-to-mesenchymal transition (EMT) [41]. SARS-CoV-2 infection has been found to disrupt host cytoskeletal homeostasis and modifications that are tightly connected to COVID-19 pathogenesis [42]. AFs and MTs participate in the syncytia formation and cell–cell fusion induced by SARS-CoV-2 infection [43]. There were distinct morphological alterations in the A549 replicon compared to those of the A549 control (Figure 10A). Cytoskeletal remodeling due to the SARS-COV-2 replicon was examined by immunofluorescence staining for actin, vimentin, and tubulin. The SARS-CoV-2 replicon induces the expression of actin, vimentin, and tubulin in replicon cells, compared to control cells, and leads to morphological changes in the replicon cells (Figure 10B–D). Vimentin expression was uniformly induced in all replicon cells in culture (Supplementary revision Figure S7).

**Figure 11:**
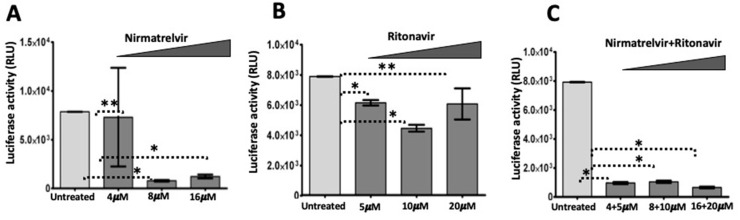
SARS-CoV-2 replication in A549 cells is sensitive to Paxlovid antiviral treatment. A549 replicon cells (1 × 10^5^) were seeded in six-well plates in triplicate in a growth medium without G-418. The next day, indicated concentrations of Nirmatrelvir or Ritonavir were added individually or in combination, and treatment was continued for an additional 72 h. The data in this figure show replicon luciferase activity as a surrogate for antiviral activity. Antiviral success was determined by Nano Luciferase activity/microgram of protein. (**A**). Concentration-dependent inhibition of replicon luciferase activity by Nirmatrelvir. (**B**). The antiviral activity of Ritonavir against SARS-CoV-2. (**C**). Combination of Nirmatrelvir plus Ritonavir (called Paxlovid) antiviral efficacy against SARS-CoV-2 replication in A549 replicon. ** *p* value > 0.05, * *p* value < 0.001, Student’s *t* test was used. Luciferases were measured three times for each scale bar (n = 3).

**A549 SARS-CoV-2 replicon cellular response to antiviral treatment:** To confirm the utility of the culture model for drug testing, A459 cells harboring the SARS-CoV-2 replicon system were subjected to treatment with FDA-approved antivirals. Testing focused on oral antiviral Paxlovid (Nirmatrelvir and Ritonavir). An IC50 of 4.4 μM was previously established for Nirmatrelvir [44,45]. Ritonavir is an oral antiretroviral drug that inhibits CYP3A and was previously used to treat HIV infection [46,47]. Ritonavir enhances the antiviral activity of Nirmatrelvir by increasing its bioavailability, thereby preventing metabolism, and has an established IC50 [48]. A549 replicon cells (1 × 10^5^) were seeded in 12-well plates, and the next day, cells were treated with increasing concentrations of Nirmatrelvir (4, 8, 16 micrograms/mL) and ritonavir (5, 10, and 20 micrograms/mL) alone and in combination using 1mL culture medium. G-418 was not used in the culture media during the antiviral testing. Cells without drug treatment served as a positive control, and wild-type A549 served as a negative control. Cells were harvested after 72 h, and the cell pellet was suspended in a lysis buffer. Nano luciferase activity was normalized with total protein concentration, and data analysis was performed as a percentage of inhibition compared to the untreated control. The antiviral assay was performed in triplicate. Each time, the luciferase values were normalized with micrograms of protein, and treatment efficacy was determined. Nirmatrelvir, the main SARS-CoV-2 antiviral, inhibited virus replication in a concentration-dependent manner (Figure 11A). There is a significant decrease in luciferase activity in the presence of Ritonavir compared to the control (Figure 11B). The effectiveness of the combined drug treatment was confirmed in the A549 replicon model (Figure 11C).

**Figure 12:**
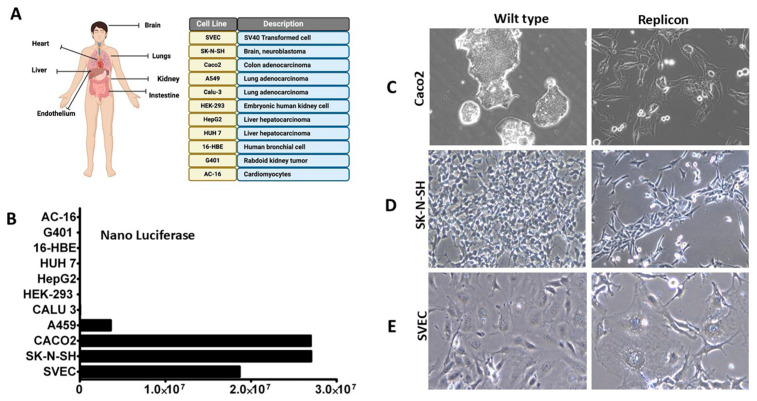
Development of a stable replicon system using multiple human cell lines. (**A**). Different human cell lines and their tissue origin tested for their ability to support replication after EV-mediated transmission of SARS-CoV-2 replicon RNA stably. (**B**). Colon adenocarcinoma (Caco2), Brain neuroblastoma epithelial cells, and SV40-transformed endothelial cells stably support SARS-CoV-2 replication. Replication of SARS-CoV-2 in A549 cells was not stable. (**C**). Light microscopy image showing that SARS-CoV-2 replicon replication in Caco2 cells induces cell shape changes, syncytia formation, and the accumulation of DMV around the nucleus. (**D**). Light microscopy image showing that SARS-CoV-2 replicon replication in neuroblastoma cell lines induces cell shape changes, syncytia formation, and the accumulation of DMV around the nucleus. (**E**). Light microscopy image showing that SARS-CoV-2 replicon replication in endothelial cells induces cell shape changes, syncytia formation, and the accumulation of DMV around the nucleus. Photographs were taken at 40× magnification.

**Human-derived cell culture models supporting stable, long-term SARS-CoV-2 sub-genomic RNA replication**: Unfortunately, SARS-CoV-2 replication in the A549 cell line is unstable, with luciferase activity diminishing over time to become negative for luciferase expression by nine months (Supplementary Figure S3). Therefore, the model was adapted to a panel of human cell lines to establish a stable replication model for the sub-genomic clone. The human cell lines were treated with cell-free supernatants collected from BHK-21 replicon (clone 8) overnight, and then the cells were cultured with media supplemented with G-418 (200 microgram/mL) (Figure 12A). Culture was monitored over an extended period to develop cell colonies. Although most lines developed antibiotic-resistant colonies after treatment with EVs derived from the BHK-21 replicon, nano luciferase expression remained very low, and replication failed to reach the robust levels observed in the A549 cell line. Colonies were selected from the other three lines, which supported the highest levels of replication and produced G-418-resistant colonies (Caco2, SK-N-SH, and SVEC) and were then developed into stable cell lines. The stable cell lines exhibited high replication and maintained high nano luciferase expression over an extended period (Figure 12B). High-level expression of the SARS-CoV-2 replicon RNA induced cytoskeletal rearrangement and cell morphological changes (Figure 12C–E). Caco-2 cells are epithelial cells derived from colon carcinoma and are used as a model for GI epithelium with apical and basal polarity. SK-N-SH are brain neuroblastoma cells, whereas SVEC is of endothelial cell origin. Cells replicating SARS-CoV-2 exhibited a larger two-dimensional profile, accompanied by cytoskeletal reorganization and cell-to-cell membrane fusion events, which are implicated in Syncytia formation.

**Figure 13:**
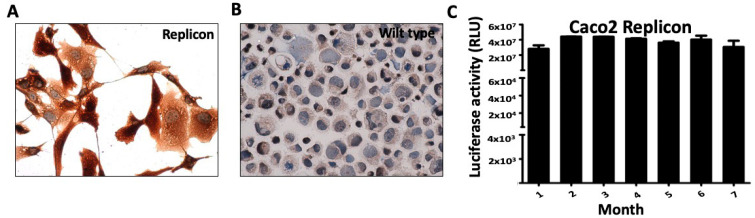
The human colon adenocarcinoma cell line (Caco-2) supports very high-level replication of SARS-CoV-2. (**A**). Expression of viral protein (Nsp1) in replicon cells by immunostaining. (**B**). Expression of viral protein (Nsp1) in control Caco2 cells. Photographs were taken at 40× magnification. (**C**). Nano luciferase activity of Caco2 replicon cell line showing that the SARS-CoV-2 replication is stable over seven months in cell culture.

The expression of viral Nsp1 in the Caco2 replicon cells was confirmed by immunostaining (Figure 13A). SARS-CoV-2 sub-genomic RNA replication in Caco2 replicon cells shows significant cytoskeletal remodeling and shape change as compared to wild-type Caco2 (Figure 13B). High-level viral replication in Caco2 cells enabled the detection of the viral Nsp1 protein by Western blotting (Supplementary Figure S4). Caco2 replicon cells show high-level SARS-CoV-2 replication, and nano luciferase expression remained stable in Caco2 over several months (Figure 13C). Similar profiles of robust SARS-CoV-2 sub-genomic RNA replication and stability were observed in the SK-N-SH and SVEC cell lines over several months (Supplementary Figure S8), with all replicon cell lines maintaining stability superior to that of the A549 line. Our results are consistent with a prior report indicating that Caco2 cells provide a robust platform for generating infectious SARS-CoV-2 virus particles [49].

**Figure 14:**
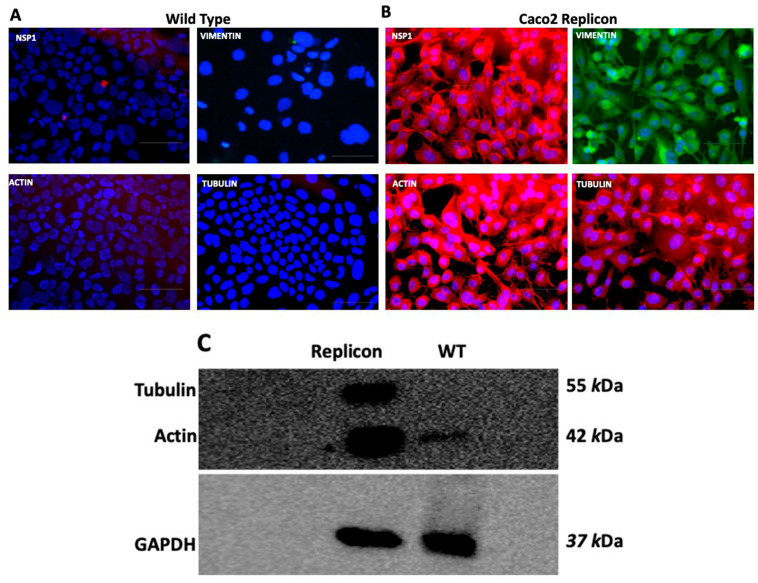
SARS-CoV-2 replication in Caco2 disrupts cell–cell adhesion and expression of genes involved in vimentin, actin, and tubulin in the cell cytoskeleton. (**A**). Immunofluorescence staining of wild-type Caco2 cells using antibodies against viral Nsp1, vimentin, actin, and tubulin. (**B**). Photographs were taken at 40× magnification. Expression of viral Nsp1, vimentin, β actin, and α tubulin in Caco2 replicon cells. (**C**). Western blot analysis demonstrates the expression of actin and tubulin in Caco2 replicon cells.

**SARS-CoV-2 RNA replication hijacks the cytoskeleton:** All coronaviruses are known to induce cytoskeletal rearrangements in both epithelial and endothelial cells, and alter intracellular trafficking, cell–cell fusion, syncytia formation, and immune dysfunction [34,42,50,51]. We examined the expression of the cytoskeletal network, comprising vimentin, actin, and tubulin, in SARS-CoV-2 replicon Caco2 and wild-type Caco2 cells using immunofluorescence. The expression of viral Nsp1, vimentin, actin, and tubulin was induced in SARS-CoV-2 replicon cells as compared to wild-type cells (Figure 14A,B). The expression of cells is dynamic and highly dependent on the differentiation status and culture conditions, which affect their function as a model for the intestinal barrier. Caco-2 cells are derived from a human colon carcinoma, but in culture, they spontaneously differentiate into enterocyte-like cells, particularly after reaching confluence. This cell line is an established model for investigating GI absorption and epithelial permeability [52] mimicking the absorptive monolayer of the small intestine. This process involves the development of microvilli and tight junctions, and the expression of brush-border-associated enzymes. The expression of actin and tubulin is low in wild-type Caco-2 cells as compared to Caco-2 replicon cells. A Western blot analysis for actin and tubulin supports the fluorescence data (Figure 14C) (**original blots are included in** Supplementary Figure S9). Caco-2 is a colonic epithelial cell known to be negative for Vimentin (a mesenchymal marker). These results suggest that SARS-CoV-2 RNA replication alone induces molecular remodeling of the cellular cytoskeleton, leading to shape changes reminiscent of EMT, cell–cell fusion, and syncytia formation.

**Figure 15:**
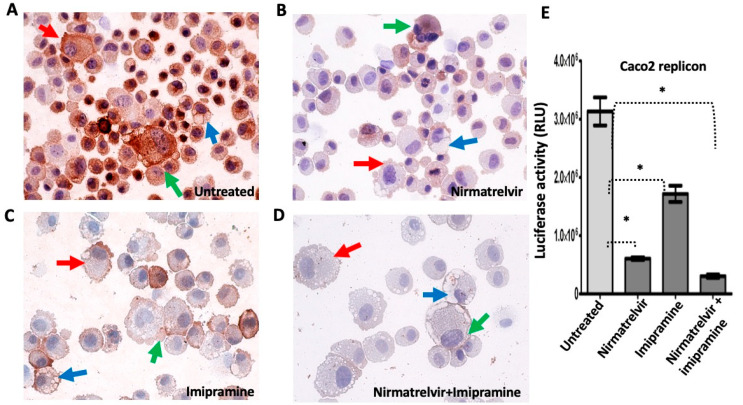
Clearing SARS-CoV-2 replication does not revert the cellular shape (red arrow), vesicular abnormalities (blue arrow), and syncytia formation (green arrow). (**A**). Immunostaining of viral Nsp1 of untreated Caco2 cells. Presence of multinucleated giant cells, and massive accumulation of DMV in the cytoplasm associated with SARS-CoV-2 RNA replication. (**B**). Nsp1 expression in Caco2 cells treated with Nirmatrelvir (16 mM) for 72 h. (**C**). Nsp1 expression levels in Caco2 replicon treated with Imipramine (10 mM) for 72 h. (**D**). Photographs were taken at 40× magnification. Nsp1 expression in Caco2 cells treated with a combination of imipramine (10 mM) and Nirmatrelvir (16 mM). (**E**). Luciferase assay showing imipramine enhances antiviral potency of Nirmatrelvir. * *p*-value < 0.001, Student’s *t*-test was used. Luciferases were measured three times for each scale bar (n = 3).

**Clearing SARS-CoV-2 replication does not revert the cellular shape, vesicular abnormalities, and syncytia formation:** Long COVID is an unexplained, multiorgan chronic disease that has been experienced by many individuals after recovery from SARS-CoV-2. The molecular basis of this newly identified infection-associated chronic condition is unknown. SARS-CoV-2 replication induces accumulation of DMV in the cytoplasm. DMVs protect viral RNA replication from innate antiviral responses and reduce the efficacy of antiviral treatment [24]. The sub-genomic replicon system in Caco-2 cells provides a functional model to test the effectiveness of Nirmatrelvir in combination with an EV inhibitor, imipramine. 1 × 10^5^ Caco2 replicon cells were seeded in multiple 12-well plates and treated with different antiviral compounds alone and with an EV inhibitor. The antiviral treatment was administered for 72 h, and treatment efficacy was assessed using a NanoLuc measurement. The model system used Caco2 cells exposed to multiple rounds of antiviral therapy with Nirmatrelvir to eliminate viral RNA replication (Supplementary Figure S5). Nirmatrelvir or Imipramine treatment alone suppressed viral RNA replication but did not result in complete elimination of viral RNA (Figure 15A–C). However, antiviral therapy in combination with EV inhibition was superior to either monotherapy, resulting in complete eradication of viral replication (Figure 15D). Combination treatment with an EV inhibitor and Nirmatrelvir shows additional antiviral success and viral clearance (Figure 15E). This finding underscores the potential of combination treatments in combating SARS-CoV-2.

**Figure 16:**
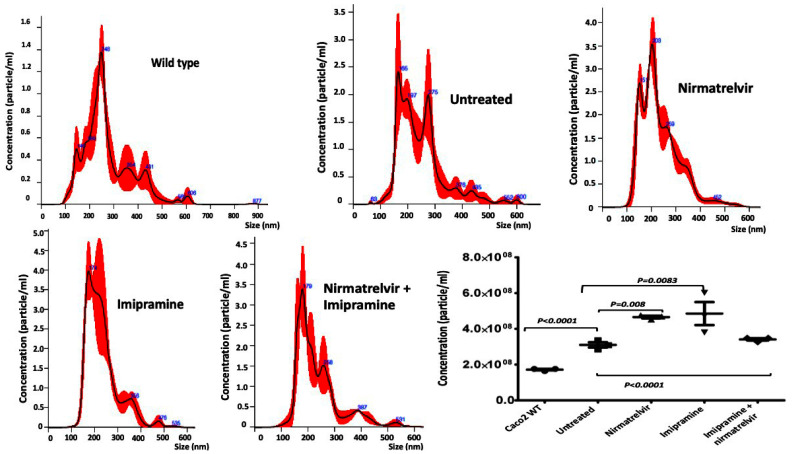
The concentration of extracellular vesicles in the supernatants of Caco-2 replicon cultured cells was measured using NTA. EV production is increased in Caco-2 replicon cells as compared to wild-type Caco-2 cells. EV production remains higher in viral-cured cells compared to wild-type Caco-2 cells.

We found that the SARS-CoV-2-induced cytoskeletal abnormalities, such as shape change, cell–cell fusion, syncytia, and vacuolation, were not reverted to normal by antiviral treatment (Supplementary Figure S6). Extracellular vesicles (EVs) are lipid membrane-bound structures released by most human cells that contain numerous biologically active compounds, including mRNA, miRNA, DNA, Lipids, and proteins. SARS-CoV-2 may hide in these vesicles and reinfect various tissues and organs of the circulatory system. The mechanism of long COVID has been attributed to the presence of an insoluble microclot resulting from hyperactivation of the inflammation and coagulation systems [53]. EVs play an essential role in inflammation and coagulopathy, which may contribute to long COVID symptoms [54,55]. We examined whether inhibiting viral replication could decrease the release and production of EVs compared to wild-type Caco2 cells. Consistent with data from human studies, SARS-CoV-2-replicating Caco2 cells release more extracellular vesicles compared to wild-type Caco2 cells, as determined by nano tracking analysis (NTA). Furthermore, eliminating viral replication does not decrease extracellular vesicle production (Figure 16). We found that imipramine alone or nirmatrelvir alone did not completely clear the virus replication. We suspect that the increase in EV production in samples treated with imipramine alone or with nirmatrelvir alone may be due to an additive effect of the virus and the drug. However, the combination treatment decreased EV production because it completely cleared virus RNA replication.

We have developed robust SARS-CoV-2 RNA replication in multiple human cells of endothelial and epithelial origin. These cell lines can be used to understand mechanisms of host-viral interaction implicated in long-term COVID and to screen antiviral drugs against SARS-CoV-2 using human cells in a BSL-2 laboratory.

## 4. Discussion

BSL-3 requirements for SARS-CoV-2 work limit our ability to understand the fundamental molecular mechanisms underlying virus–host interactions, particularly the reversible and irreversible cellular responses associated with SARS-CoV-2 replication. Self-replicating RNAs, known as replicons, serve as an ideal model system to study pathogenic RNA viruses. Developing a stable replicon system for SARS-CoV-2 in human cells has been challenging due to the virus’s large genome and the cytotoxicity associated with its replication. Many laboratories have attempted to develop a stable replication system for SARS-CoV-2 in human cells but have failed due to the virus’s intrinsic cytotoxicity [56,57,58,59,60]. Liu et al. developed an improved SARS-CoV-2 replicon RNA by introducing two-point mutations in the viral Nsp1 at residues K164A/H165A, which reduced interactions between the host ribosome and the C-terminus of Nsp1, thereby increasing host protein translation [28]. These two-point mutations also decreased the cytotoxicity associated with Nsp1, allowing for the selection of stable clones that produce SARS-CoV-2 subgenomic RNA. The BHK-21 replicon system can be maintained in a BSL-2 setting and passaged multiple times. The high-level replication of SARS-CoV-2 in BHK-21 replicon cells allows for the measurement of viral proteins by Western blotting and immunocytochemical methods. We found that these cells exhibit several features, including massive cytoskeletal rearrangements, shape changes, the accumulation of double-membrane vesicles, syncytia formation, and cell–cell fusion [42,43]. Replicon cells shed a high number of DMVs/EVs compared to wild-type cells.

Our previous work demonstrates that EVs are a crucial component in maintaining cellular homeostasis during RNA virus replication [11]. EVs containing viral RNA and RNA-replicative intermediates are released during persistent viral replication to maintain cellular homeostasis and virus persistence in cell culture. This study examined whether EVs derived from BHK-21 replicon cells could transfer replication-competent SARS-CoV-2 RNA to establish a functional and stable replicon system in human cells. Our data show that EV depletion is unable to transfer replicon RNA, and cells did not develop G-418-resistant cell clones. The results show that replication-competent SARS-CoV-2 RNA can be transferred through EVs, in a virion-independent manner, to permissive human cells, triggering protein production and viral RNA replication. These events do not require infectious virus particles since the BHK-21 SARS-CoV-2 replicon cells lack the viral structural genes. The results indicate that EV-mediated transfer of viral RNA is a crucial mechanism for viral RNA transmission, thereby contributing to viral spread and immune surveillance. The human replicon system, developed in epithelial and endothelial cells, will enable us to understand virus–host interactions at the molecular level.

The mechanisms of the EV uptake by human cells are unclear. This study did not investigate the uptake mechanism since EVs are taken up by recipient mammalian cells through various mechanisms, with the specific pathway depending on factors such as EV composition, recipient cell type, and the surrounding microenvironment. The primary modes of uptake involve multiple forms of endocytosis and direct fusion with the cell’s plasma membrane. The EVs are taken up by a variety of endocytic pathways, including clathrin-dependent endocytosis and clathrin-independent pathways such as caveolin-mediated uptake, macropinocytosis, phagocytosis, and lipid raft–mediated internalization [61,62,63]. The uptake mechanism used by a given EV may depend on proteins and glycoproteins found on the surface of both the vesicle and the target cell. Some EVs can fuse directly with the plasma membrane of the recipient cell, delivering their cargo into the cytoplasm without being enclosed in an endocytic vesicle. This process involves the physical merging of the EV’s lipid bilayer with the cell’s plasma membrane. We found that EV uptake by human cells is highly efficient, as nearly all cells showed DiD-red fluorescence. The data presented in this paper show that EV-mediated transfer of viral RNA is an efficient process.

Although SARS-CoV-2 primarily infects lung epithelial cells, we found that SARS-CoV-2 replicates in A549 cells. Our results show that replicating A549 cells do not stably harbor SARS-CoV-2 replication. These cells frequently lose the ability to reproduce, becoming permanently incapable of adapting to virus replication. An ideal in vitro system for antiviral screening should possess characteristics that facilitate easy growth, maintenance, and passaging while allowing for high-titer viral replication. Therefore, multiple human cell lines were tested to identify one that is well adapted to long-term SARS-CoV-2 RNA replication. Only a select group of human cell lines were both susceptible and supported stable SARS-CoV-2 RNA replication, with lung-derived cell lines (A549, CALU-3, and 16-HBE) failing to support or sustain viral RNA replication. Robust and stable viral RNA replication was achieved in gastrointestinal, neuronal, and vascular endothelial cell lines, resulting in viral protein expression and morphological changes compared with wild-type cells. The physiological properties that enable stable SARS-CoV-2 RNA replication in these lines, compared to those derived from the lungs, remain unclear.

Our study confirms that SARS-CoV-2 RNA replication induces massive cytoskeletal rearrangements, morphological changes, cell membrane fusion, and syncytia formation, resulting in the fusion of neighboring cells [35]. These findings are consistent with post-mortem samples from individuals who died of severe COVID-19, which show the presence of multinucleated pneumocytes expressing viral RNA and proteins [64]. The cytoskeleton is an intricate network that controls cell shape, cargo transport, signal transduction, and cell division. Our study agrees with many studies published in high-impact journals that have demonstrated the essential roles of the cytoskeleton in mediating the outcome of host-virus interactions [34,65,66]. Ralf Bartenschlager’s group and other researchers have shown that SARS-CoV-2 infection induces cytoskeletal proteins [67,68]. The EV-derived replicon system effectively reproduced these cytoskeletal abnormalities. DMV, the virus-induced organelle supporting replication, was observed around the nuclear membrane in all three stable replicon cell lines, consistent with those observed in tissues infected with SARS-CoV-2 and other coronaviruses [64]. These publications demonstrate SARS-CoV-2 replication in DMV [69,70,71]. Some of these publication claims that all RNA viruses use similar strategies to replicate in the cytoplasm.

The formation of EVs and DMVs relates to a cellular process of vesiculation facilitated by cell-derived membranes [25]. Vesicle formation consists of two steps: membrane deformation (wrapping a membrane around a substance) and membrane scission (pinching off the vesicles from the larger body of membrane). The process of vesicle biogenesis is integrated with cellular lipid metabolism. Viral replication and assembly are also intimately linked with membrane lipid bilayers. The results show that inhibiting EV trafficking reduces SARS-CoV-2 RNA replication. The replicon system recapitulated sensitivity to targeted antivirals, supporting its role in future antiviral screening. These data are consistent with the idea that EV plays an essential role in RNA virus replication, since inhibiting EV trafficking activates an innate antiviral response that induces interferon production [11,72].

We found that the viral cure in the stable replicon model did not reverse extracellular vesicle production. SARS-CoV-2 RNA replication reprograms the cellular cytoskeleton, causing a massive accumulation of double-membrane vesicles. The virus-induced cellular reprogramming is not reversed after viral cure. The reason for the persistent EV production after viral cure is unknown. We propose that viruses induce the production of extracellular vesicles (EVs) by hijacking the cell’s normal EV biogenesis pathways, leading to the packaging of viral components, including proteins, RNA, and even whole virions, into these vesicles. This process often involves direct interactions between viral proteins and host EV machinery, such as the ESCRT complex and autophagy, which are essential for forming the internal structures of EVs. The resulting virus-modified EVs can then be released and function to propagate the infection, alter host immune responses, or facilitate viral evasion of immune detection. This phenomenon challenges the traditional view of viral infection as a purely transient event. Viruses can utilize extracellular vesicles (EVs) as a “Trojan horse” to transport viral RNA to distant tissues and inflammatory cells, and to re-engage with them, potentially contributing to the development of long-term symptoms. These results suggest that post-viral illness can lead to lasting cellular abnormalities even after the acute infection resolves. The chronic symptoms of conditions like long COVID and Myalgic Encephalomyelitis/Chronic Fatigue Syndrome (ME/CFS) are linked to sustained immune dysregulation, persistent mitochondrial dysfunction, and other cellular damage.

Our studies using the replicon model show SARS-CoV-2 replication induces cytoskeletal abnormalities. Cytoskeletal abnormalities are linked to a wide range of human diseases, including neurodegenerative disorders like Alzheimer’s and Parkinson’s, various cancers, and some cardiovascular and skin conditions. These defects disrupt fundamental cellular processes, including transport, shape, and movement, leading to cellular dysfunction and the development of pathological conditions. For example, disruptions in neuronal transport due to cytoskeletal defects are a hallmark of many neurodegenerative diseases. Future work should focus on understanding the molecular biology of host cell reprogramming, the cytoskeletal abnormalities induced during SARS-CoV-2 infection, and their implications for human health.

## 5. Conclusions

We have developed stable cell clones derived from lung epithelial cells, gastrointestinal epithelial cells, neuronal cells, and endothelial cells that replicate SARS-CoV-2 sub-genomic RNA. Our preliminary data indicate that only select cell types (Lungs, GI, Brain, and endothelium) can sustain robust virus replication and induce syncytia formation, cytoskeletal remodeling, EMT, and DMV accumulation. These models can be used to investigate the virus-induced cellular programming and its reversal after viral cure. SARS-CoV-2 replicon cell lines are non-infectious, self-replicating platforms that provide a safe and effective tool for studying virus replication mechanisms and the development of antiviral treatments. Because they lack the structural genes needed to produce infectious virus particles (virions), these modified systems can be handled in lower-level biosafety facilities (BSL-2) rather than in the BSL-3 facilities required for live, infectious SARS-CoV-2. The integration of reporter genes, such as luciferase, enables the rapid and straightforward quantification of viral replication. By measuring the reporter’s signal, researchers can screen thousands of compounds to find a potential inhibitor. Scientists can use replicon systems in genetically modified cells to study how the virus interacts with host proteins. For example, testing a replicon in knockout cell lines can confirm if a particular host factor is essential for viral replication. The improved safety and accessibility of replicon cell lines enable more academic and pharmaceutical laboratories to contribute to research on SARS-CoV-2.

## Data Availability

The original contributions presented in this study are included in this article/Supplementary Materials. Further inquiries can be directed to the corresponding author. https://libguides.tulane.edu/datamanagement/datasharing (accessed on 18 January·2026).

## Supplementary Materials

The following supporting information can be downloaded at: https://www.mdpi.com/article/10.3390/v18010145/s1, Figure S1: Original Western blot showing the detection of SARS-CoV-1 Nsp1 and GAPDH in wild type BHK-21 cells and BHK-21 replicon clone 8 shown in Figure 2B; Figure S2: Immunofluorescence assay of A549 replicon cells showing imipramine, calpeptin and ketotifen treatment inhibits viral Nsp1 expression. Antibody dilution used 1:1000. Photographs were taken at 40× magnification; Figure S3: Measurement of nano luciferase activity of SARS-CoV-2 replicon in A549 cells over 9 months. Replication of SARS-CoV-2 is not stable in A549 cells; Figure S4: Original Western blot for detection of viral Nsp1 and GAPDH protein expression in wild type Caco2 cells and Caco2 SARS-CoV-2 replicon shown in Figure 12C; Figure S5: The antiviral success of Nirmatrelvir in Caco2 replication measured by Luciferase assay. Replicon cells were sensitive to multicycle Nirmatrelvir treatment; Figure S6: Hematoxylin staining of cytospin slides showing that inhibition of SARS-CoV-2 replication does not reverse the virus-induced cellular abnormalities. (A). Untreated Caco2 replicon. (B). Nirmatrelvir-treated replicon. (C). Imipramine-treated replicon. (D). Combination treatment of Nirmatrelvir plus imipramine. Photographs were taken at 40× magnification; Figure S7: Vimentin staining of A549 replicon cells representing the data shown in Figure 10B. Photographs were taken at 40× magnification; Figure S8: Stable luciferase expression in SK-N-SH and SVEC SARS-CoV-2 replicon cells; Figure S9: Unmodified original Western blot of Figure 14C.

## Abbreviations

The following abbreviations are used in this manuscript: EVs, extracellular vesicles; BHK-21 Baby Hamster Kidney; SVEC, SV40 transformed mouse endothelial cell line; TEM, transmission electron microscopy; DMVs, double membrane vesicles; G-418, geneticin sulfate, SARS-CoV-2, severe acute respiratory syndrome coronavirus 2; MVs, microvesicles; CMs, convoluted membranes; DMSs, double-membrane spherules; sg mRNAs, sub-genomic mRNA transcription; BSL-3, biosafety level 3; IBC, Tulane Institutional Biosafety Committee; SLVHCS, Southeast Louisiana Veterans Health Care System; NTA, nanoparticle tracking analysis; LSM, light scattered mode; MFI, mean fluorescence intensity; TBS, tris-buffered saline; RT, room temperature; RLU, relative light units; Nano Luc, nano luciferase; A549, human lung alveolar epithelial cells; aSMase, sphingomyelinase; AFs, actin filaments; MTs, microtubules; IFs, intermediate filaments; TNTs, tunneling nanotubes; GFAP, glial fibrillary acidic protein; EMT, epithelial-to-mesenchymal transition; Caco2, colon adenocarcinoma.
