# Supplementary material for: Virion-Independent Extracellular Vesicle (EV)-Dependent Transmission of SARS-CoV-2 as a Potential New Mechanism of Viral RNA Spread in Human Cells
Source: Viruses. 2026 Jan 22;18(1):145. doi: 10.3390/v18010145 (PMC12846536; doi:10.3390/v18010145)
Supplement: Supplementary file 1 [file viruses-18-00145-s001.zip › viruses-3804092-supplementary files.pdf]

Article

Virion-Independent Extracellular Vesicle (EV)-Dependent Transmission of SARS-CoV-2 as a Potential New Mechanism of Viral RNA Spread in Human Cells

Nergiz Ekmen [1], Ali Riza Koksal [2], Dong Lin [2], Di Tian [2], Paul Thevenot [3], Sarah Glover [1] and Srikanta Dash [1,2,4,*]

1 Division of Gastroenterology and Hepatology, Tulane University Health Sciences Center, New Orleans, LA 70112, USA; nekmen@tulane.edu
2 Department of Pathology and Laboratory Medicine, Tulane University Health Sciences Center, 1430 Tulane Avenue, New Orleans, LA 70112, USA; akoksal@tulane.edu (A.R.K.); dlin6@tulane.edu (D.L.); dtian2@tulane.edu (D.T.); sglover3@tulane.edu (S.G.)
3 Department of Gastroenterology and Hepatology, Institute of Translational Research, Ochsner Health, New Orleans, LA,70121, USA; paul.thevenot@ochsner.org
4 Southeast Louisiana Veterans Health Care System, 2400 Canal Street, New Orleans, LA 70119, USA
* Correspondence: sdash@tulane.edu; Tel.: +1-504-988-2519; Fax: +1-504-988-7389

Supplementary Files

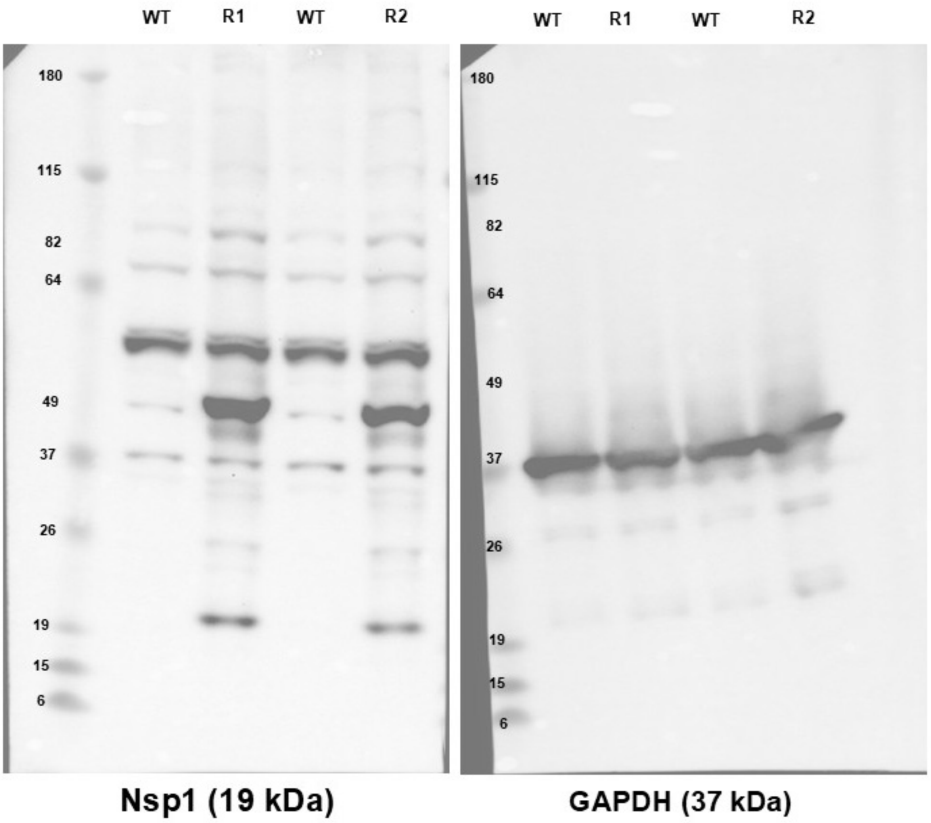

Supplemental Figure 1. Original Western blot showing the detection of SARS-CoV-1 Nsp1 and GAPDH in wild type BHK-21 cells and BHK-21 replicon clone 8 shown in Figure 2B.

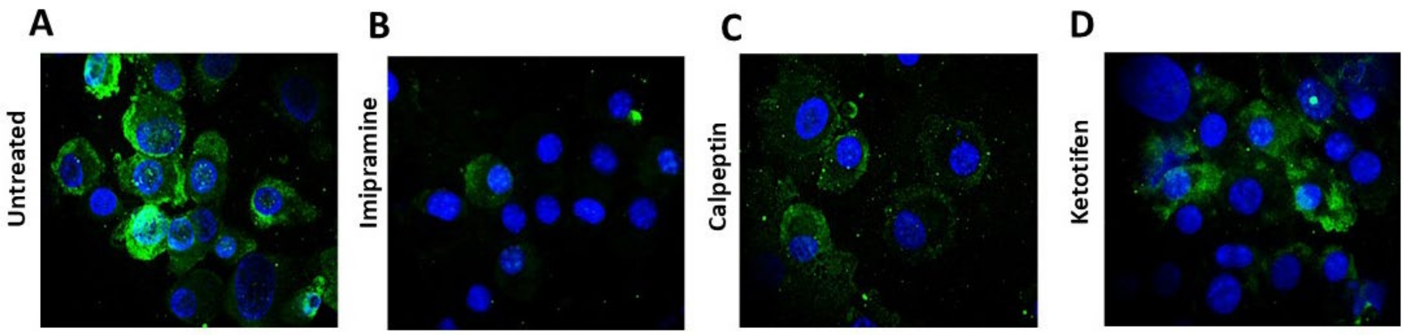

Supplemental Figure 2. Immunofluorescence assay of A549 replicon cells showing imipramine, calpeptin and ketotifen treatment inhibits viral Nsp1 expression. Antibody dilution used 1:1000. Photographs were taken at 40x magnification.

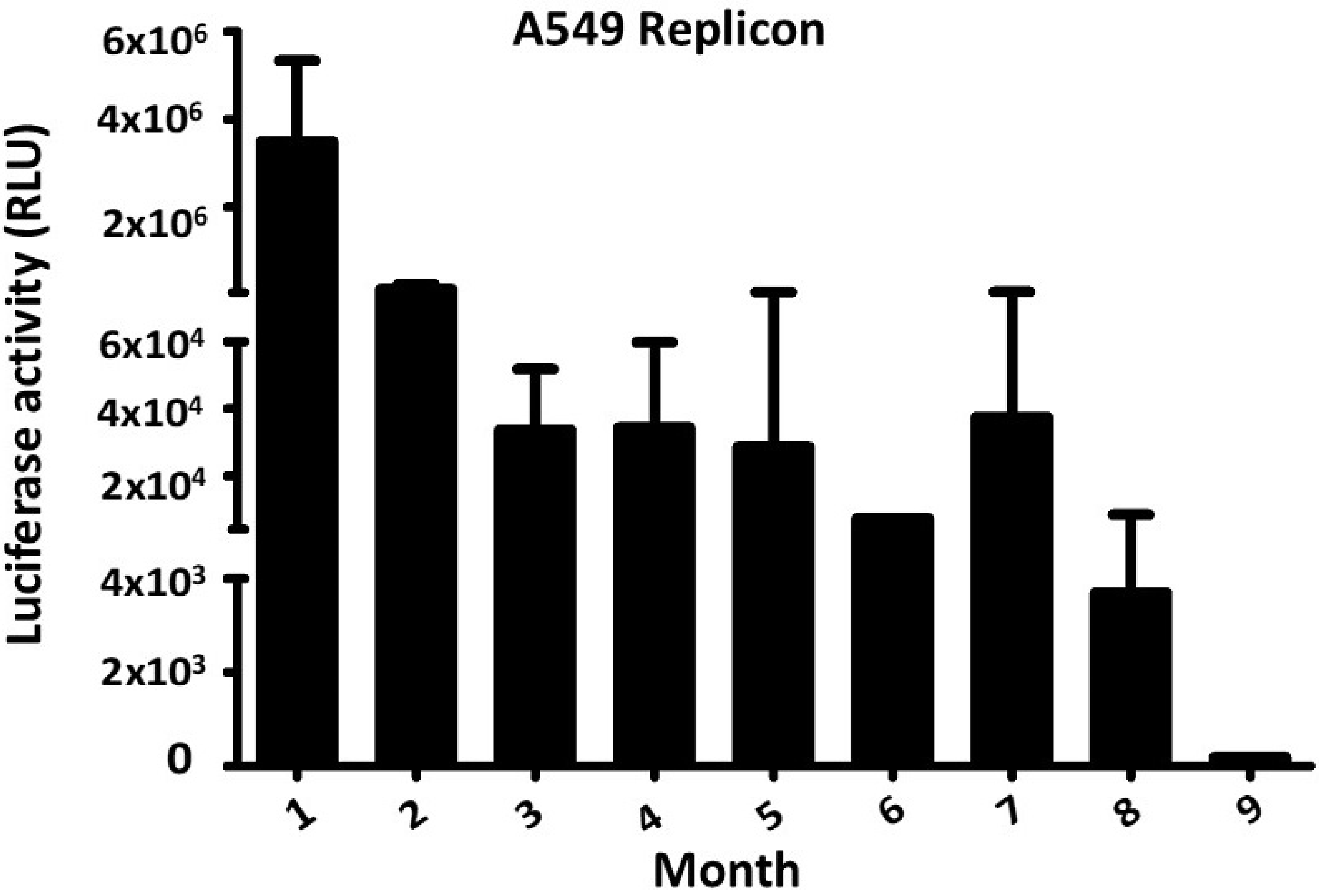

Supplemental Figure 3. Measurement of nano luciferase activity of SARS-CoV-2 replicon in A549 cells over 9 months. Replication of SARS-CoV-2 is not stable in A549 cells.

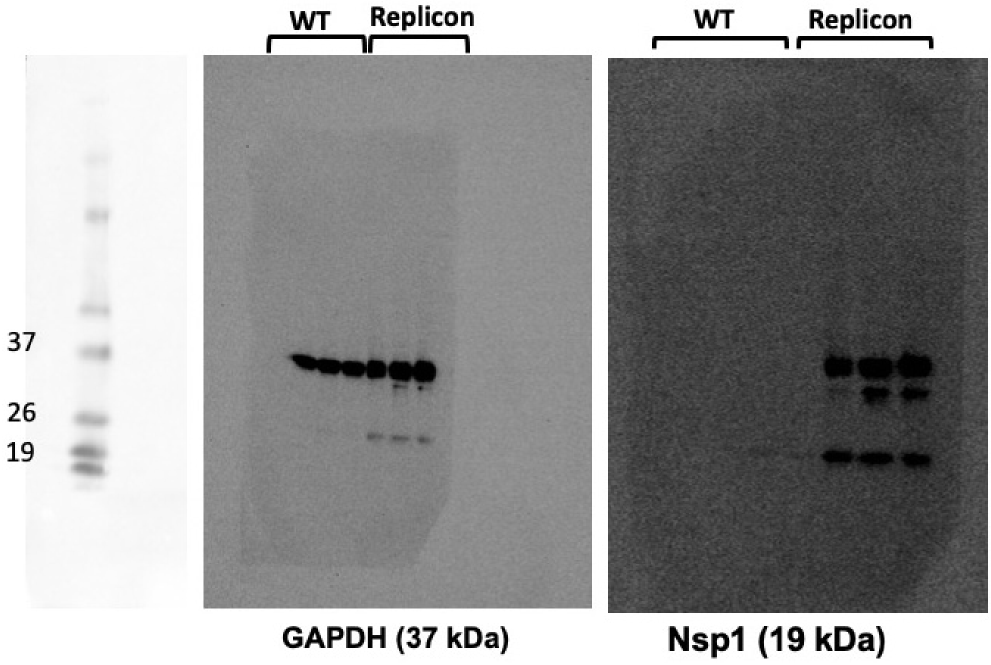

Supplemental Figure 4. Original Western blot for detection of viral Nsp1 and GAPDH protein expression in wild type Caco2 cells and Caco2 SARS-CoV-2 replicon shown in Figure 12C.

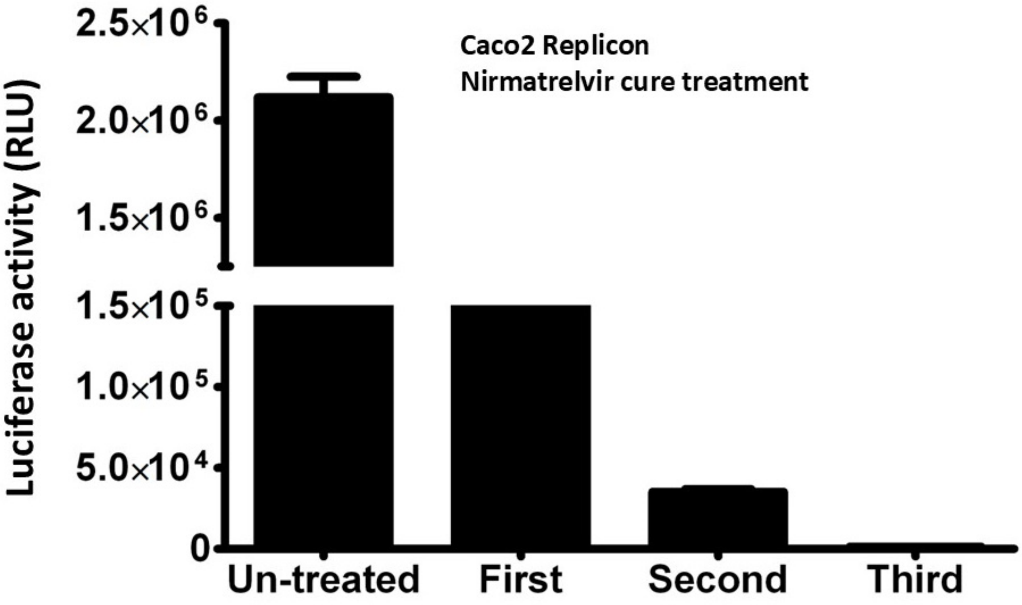

Supplemental Figure 5. The antiviral success of Nirmatrelvir in Caco2 replication measured by Luciferase assay. Replicon cells were sensitive to multicycle Nirmatrelvir treatment.

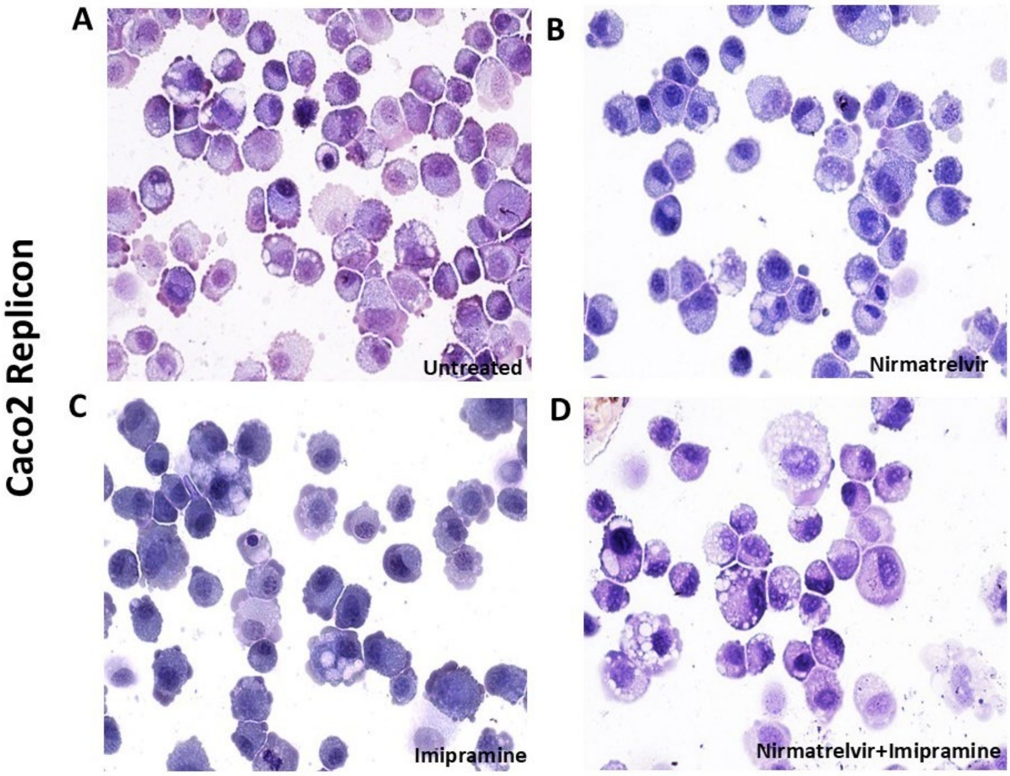

Supplemental Figure 6. Haematoxylin staining of cytospin slides showing that inhibition of SARS-CoV-2 replication does not reverse the virus-induced cellular abnormalities. (A). Untreated Caco2 replicon. (B). Nirmatrelvir treated replicon. (C). Imipramine treated replicon. (D). Combination treatment of Nirmatrelvir plus imipramine. Photographs were taken at 40x magnification.

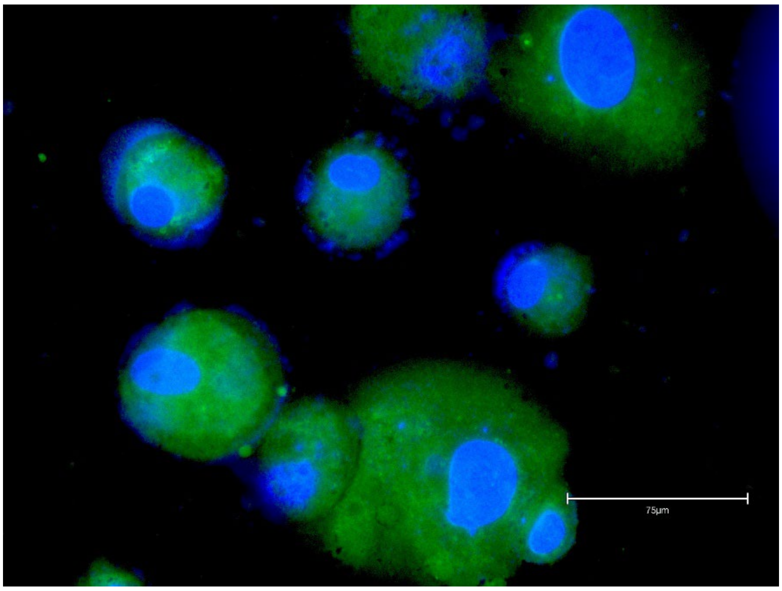

Supplemental Figure 7: Vimentin staining of A549 replicon cells representing the data shown in Figure 10B. Photographs were taken at 40x magnification.

viruses

MDPI

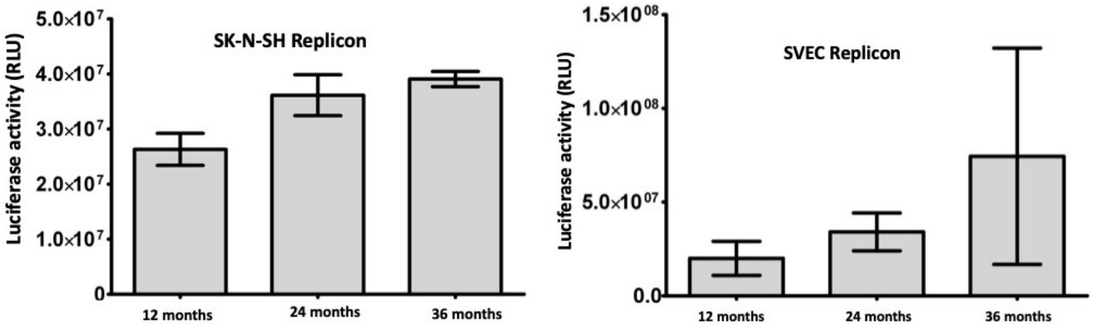

Supplemental Figure 8: Stable luciferase expression in SK-N-SH and SVEC SARS-CoV-2 replicon cells.

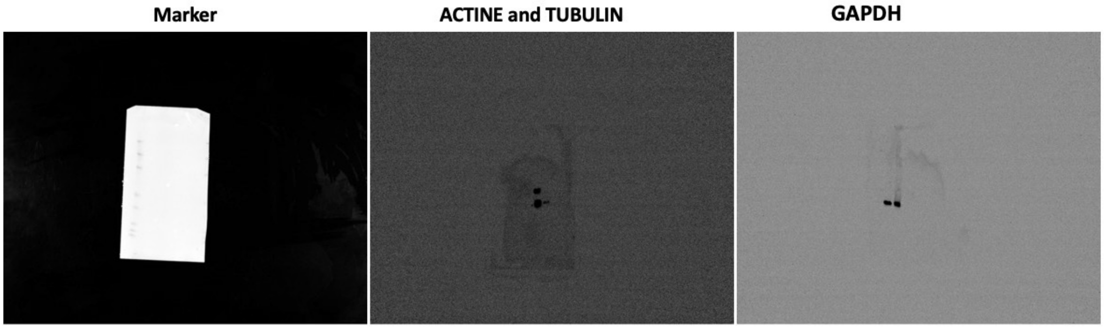

Supplementary Figure 9: Unmodified Original Western blot of Figure 14 C
