# Supplementary figures and images for: Virion-Independent Extracellular Vesicle (EV)-Dependent Transmission of SARS-CoV-2 as a Potential New Mechanism of Viral RNA Spread in Human Cells
Source: Viruses. 2026 Jan 22;18(1):145. doi: 10.3390/v18010145 (PMC12846536; doi:10.3390/v18010145)

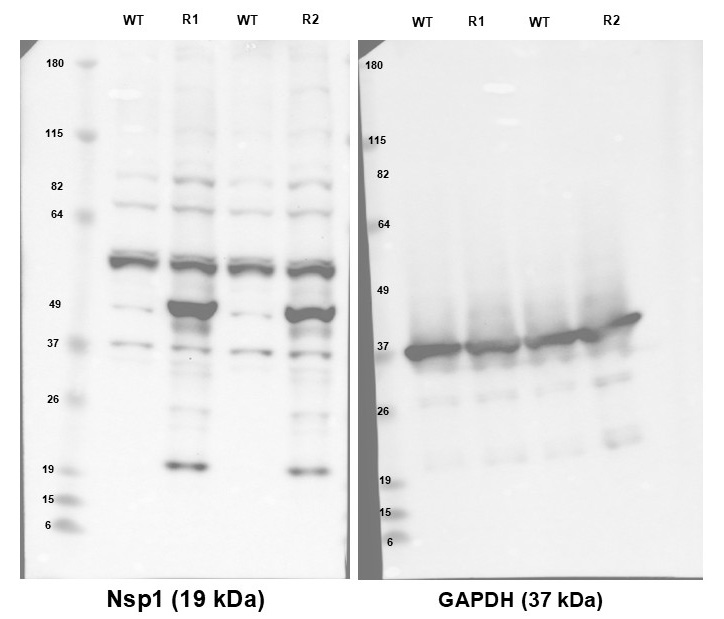

Supplement: Supplementary file 1 [file viruses-18-00145-s001.zip › Supp.figure_1.jpg]

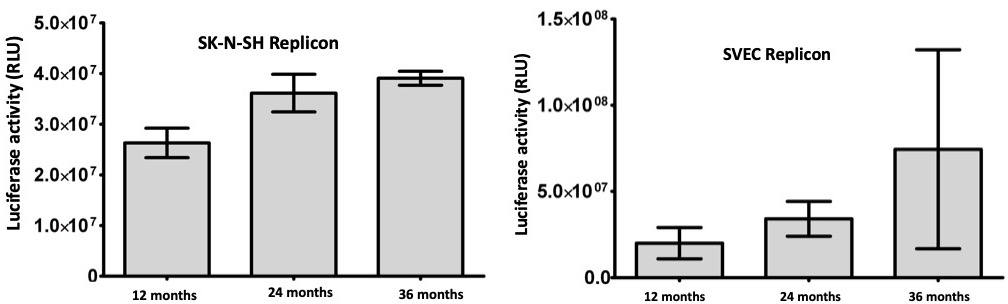

Supplement: Supplementary file 1 [file viruses-18-00145-s001.zip › Supp.figure_8.jpg]

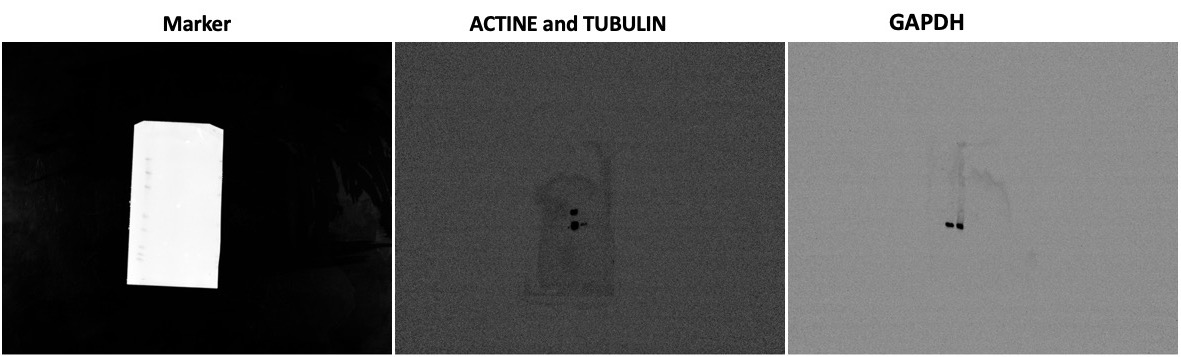

Supplement: Supplementary file 1 [file viruses-18-00145-s001.zip › Supp_figure 9_.jpg]

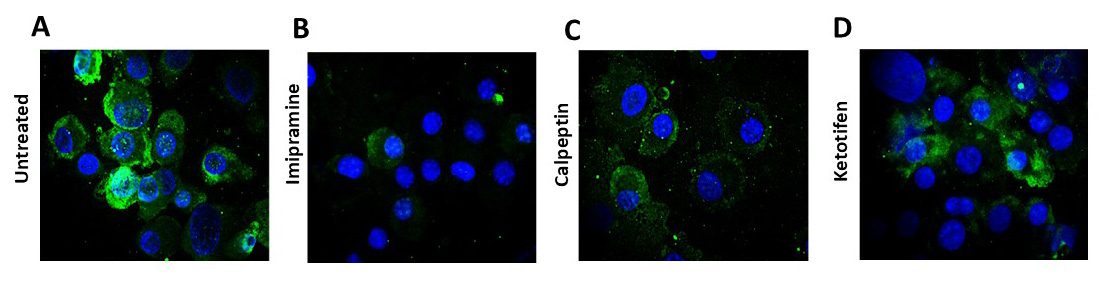

Supplement: Supplementary file 1 [file viruses-18-00145-s001.zip › Supp_figure_2.jpg]

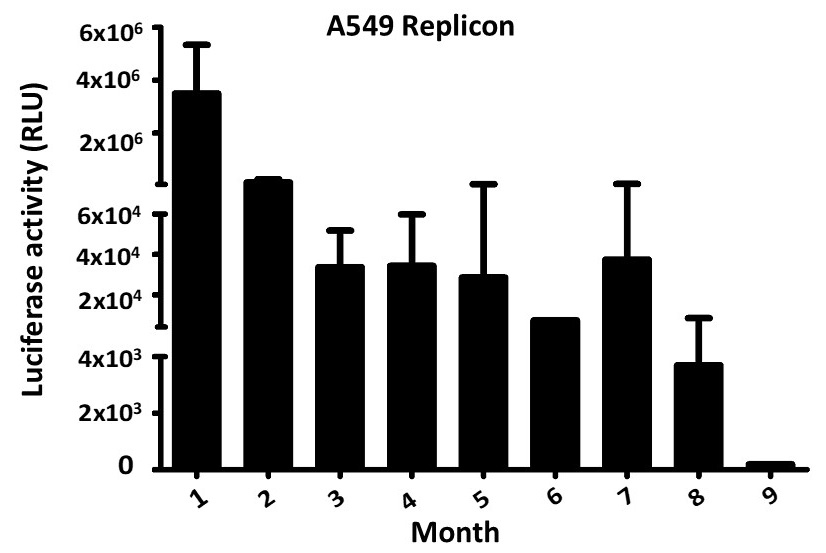

Supplement: Supplementary file 1 [file viruses-18-00145-s001.zip › Supp_figure_3.jpg]

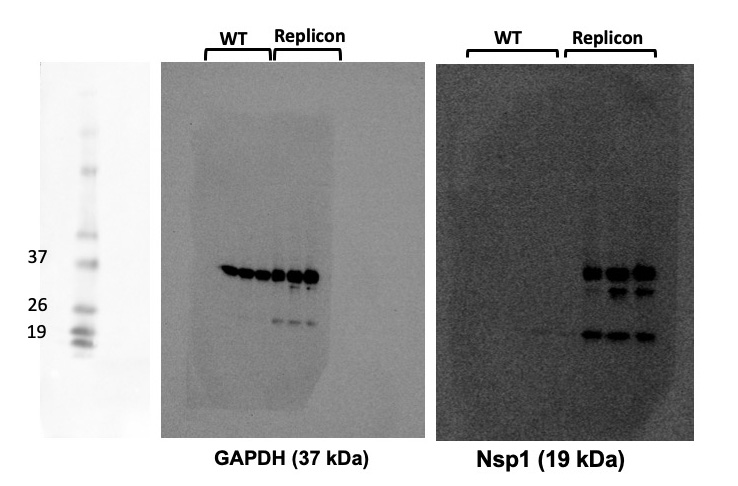

Supplement: Supplementary file 1 [file viruses-18-00145-s001.zip › Supp_figure_4.jpg]

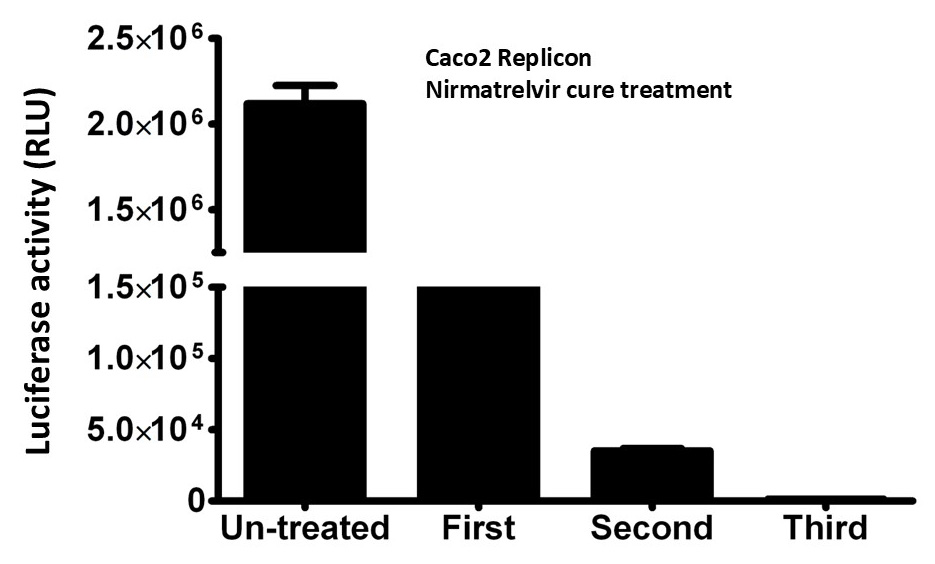

Supplement: Supplementary file 1 [file viruses-18-00145-s001.zip › Supp_figure_5.jpg]

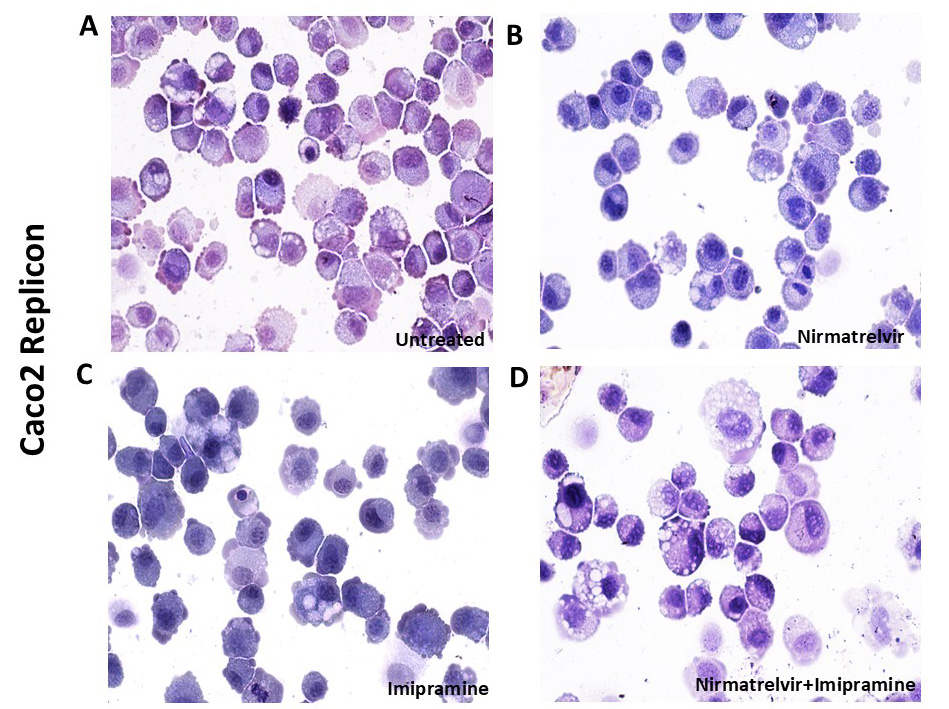

Supplement: Supplementary file 1 [file viruses-18-00145-s001.zip › Supp_figure_6.jpg]

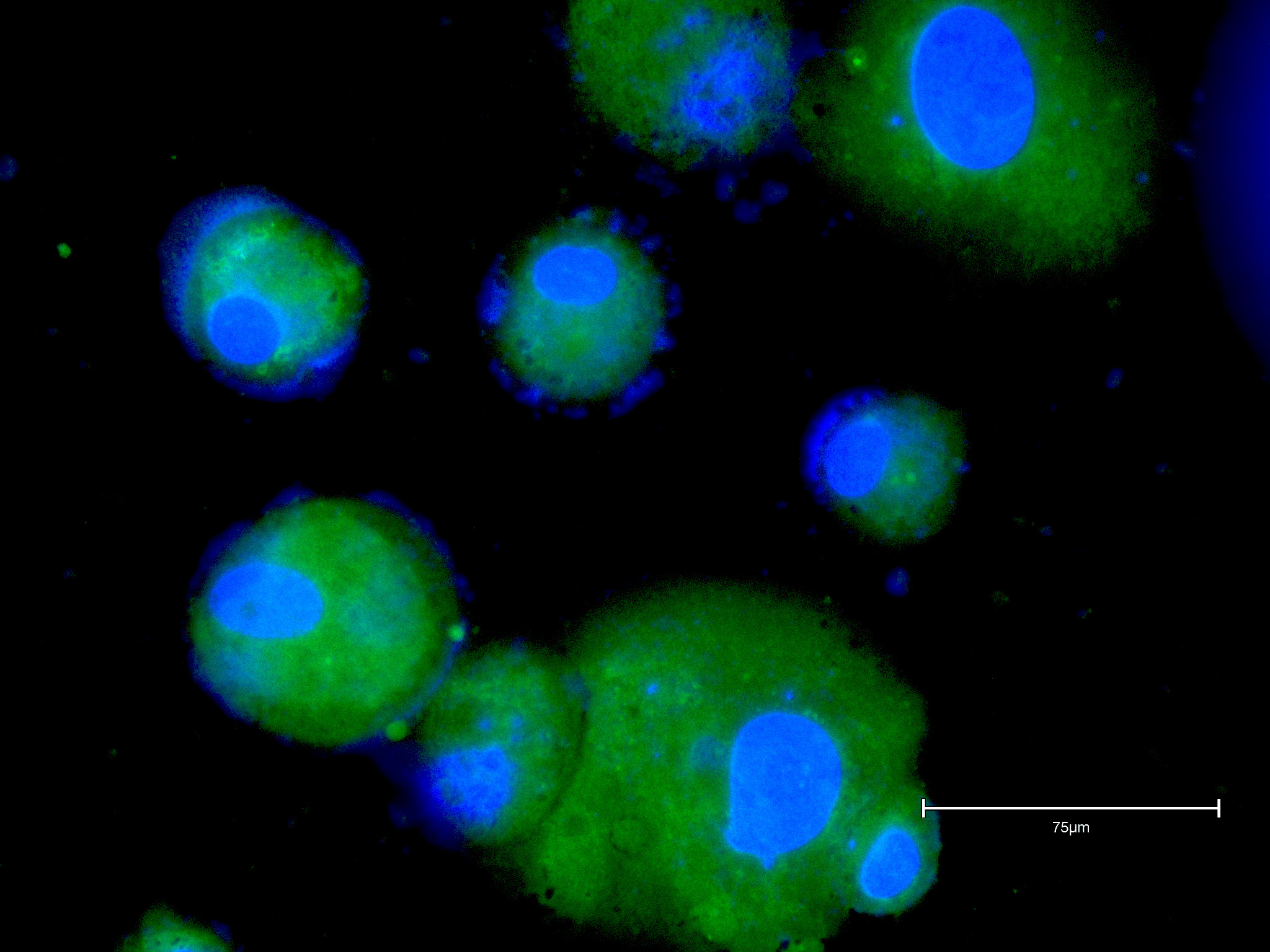

Supplement: Supplementary file 1 [file viruses-18-00145-s001.zip › Supp_figure_7.jpg]
